# The genus *Chrysanthemum*: Phylogeny, biodiversity, phytometabolites, and chemodiversity

**DOI:** 10.3389/fpls.2022.973197

**Published:** 2022-08-11

**Authors:** Da-Cheng Hao, Yanjun Song, Peigen Xiao, Yi Zhong, Peiling Wu, Lijia Xu

**Affiliations:** ^1^School of Environment and Chemical Engineering, Biotechnology Institute, Dalian Jiaotong University, Dalian, China; ^2^Institute of Molecular Plant Science, University of Edinburgh, Edinburgh, United Kingdom; ^3^Institute of Medicinal Plant Development, Chinese Academy of Medical Sciences and Peking Union Medical College, Beijing, China; ^4^Key Laboratory of Bioactive Substances and Resources Utilization of Chinese Herbal Medicine, Ministry of Education, Beijing, China

**Keywords:** *Chrysanthemum*, *Chrysanthemum morifolium*, phylogenetic relationship, phytochemistry, chemodiversity, pharmacophylogeny

## Abstract

The ecologically and economically important genus *Chrysanthemum* contains around 40 species and many hybrids and cultivars. The dried capitulum of *Chrysanthemum morifolium* (CM) Ramat. Tzvel, i.e., *Flos Chrysanthemi*, is frequently used in traditional Chinese medicine (TCM) and folk medicine for at least 2,200 years. It has also been a popular tea beverage for about 2,000 years since Han Dynasty in China. However, the origin of different cultivars of CM and the phylogenetic relationship between *Chrysanthemum* and related Asteraceae genera are still elusive, and there is a lack of comprehensive review about the association between biodiversity and chemodiversity of *Chrysanthemum*. This article aims to provide a synthetic summary of the phylogeny, biodiversity, phytometabolites and chemodiversity of *Chrysanthemum* and related taxonomic groups, focusing on CM and its wild relatives. Based on extensive literature review and in light of the medicinal value of chrysanthemum, we give some suggestions for its relationship with some genera/species and future applications. Mining chemodiversity from biodiversity of *Chrysanthemum* containing subtribe Artemisiinae, as well as mining therapeutic efficacy and other utilities from chemodiversity/biodiversity, is closely related with sustainable conservation and utilization of Artemisiinae resources. There were eight main cultivars of *Flos Chrysanthemi*, i.e., Hangju, Boju, Gongju, Chuju, Huaiju, Jiju, Chuanju and Qiju, which differ in geographical origins and processing methods. Different CM cultivars originated from various hybridizations between multiple wild species. They mainly contained volatile oils, triterpenes, flavonoids, phenolic acids, polysaccharides, amino acids and other phytometabolites, which have the activities of antimicrobial, anti-viral, antioxidant, anti-aging, anticancer, anti-inflammatory, and closely related taxonomic groups could also be useful as food, medicine and tea. Despite some progresses, the genetic/chemical relationships among varieties, species and relevant genera have yet to be clarified; therefore, the roles of pharmacophylogeny and omics technology are highlighted.

## Introduction

**Figure 1:**
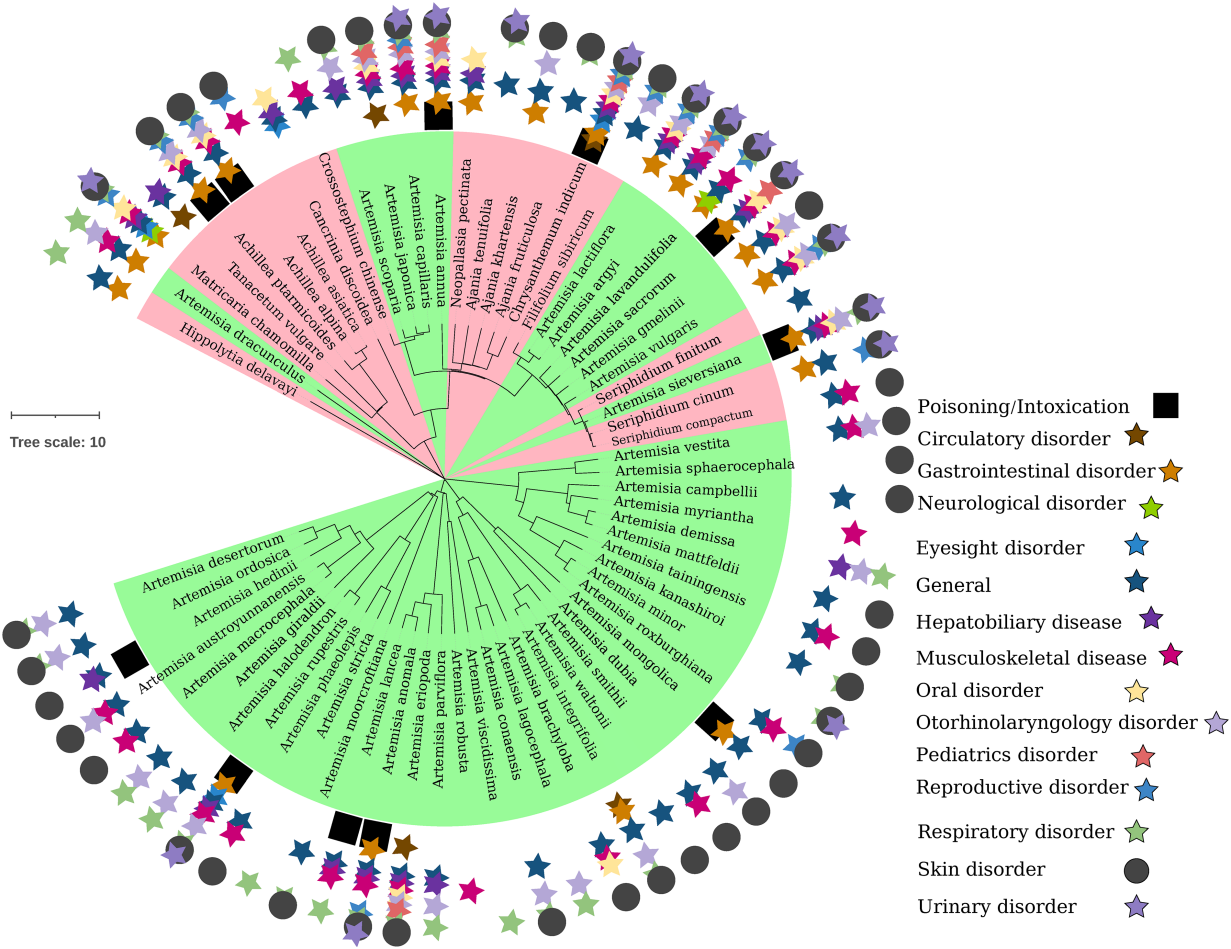
Phylogenetic relationship of Artemisiinae species, including *Chrysanthemum* and its wild relatives. The distribution of therapeutic efficacy of ethnomedicinal species on the phylogenetic tree of Chinese taxa is shown. In the inner circle, *Artemisia* species are in green area, and non-*Artemisia* species are in red area. From the inside to the outside of the outer circle are poisoning, circulatory diseases, gastrointestinal diseases, nervous system diseases, eye diseases, other/general diseases, hepatobiliary diseases, musculoskeletal diseases, oral diseases, ear/nose/throat diseases, pediatric diseases, reproductive system diseases, respiratory diseases, skin diseases, and urinary diseases, indicated by stars of different colors, square or circle.

Anthemideae is a taxonomically controversial Asteraceae (Compositae) tribe, in which phylogenetic relationships are still not settled (Criado Ruiz et al., 2021). In Anthemideae, Artemisiinae and Matricariinae are the two most widely distributed subtribes (Watson et al., 2002; Shen et al., 2021). Artemisiinae (Figure 1) includes 1/3 species of tribe Anthemideae, consisting of more than 600 species of 18–19 genera, which are mainly in the northern hemisphere, especially in Central Asia and East Asia. *Chrysanthemum*, a small Artemisiinae genus, is native to East Asia and northeastern Europe. It contains around 40 species and many hybrids and cultivars, most of which originate from East Asia, and China is the center of diversity (Liu et al., 2012). Innumerable horticultural varieties and cultivars of *Chrysanthemum* exist. The *Chrysanthemum* plants are perennial herbs. Their leaves are undivided or palmately/pinnately divided once or twice. The capitulum is heteromorphic, solitary at the top of the stem, a few or more of which are arranged into corymbose or compound corymbose inflorescences at the top of stem and branch. The marginal flowers are female, tongue shaped, 1-layer (multi-layer in cultivated varieties), and the central disk flowers are bisexual and tubular. The involucre is shallowly discoid, rarely campanulate. The involucral bracts are 4–5 layers, with white, brown, black brown or brownish black edges. The receptacle is protuberant, hemispherical or conical, without stipules. The tongue shaped flowers are yellow, white or red, and the tongue can be as short as 1.5 mm, and as long as 2.5 cm or longer. All tubular flowers are yellow, with five teeth at the top. The style branches are linear, apically truncate. The anther base is obtuse, and apical appendages are lanceolate ovate or oblong. All achenes are isomorphic, nearly cylindrical and narrowed to the lower part, with 5–8 longitudinal veins and no coronal crown hair.

As the most remarkable taxon of *Chrysanthemum*, *Chrysanthemum morifolium* Ramat. Tzvel (CM, known as “Ju Hua” in Chinese) originated in China as a medicinal, food and ornamental plant. Its history began about 3,000 years ago, since Xia and Shang Dynasties (Zhang and Dai, 2009). CM was introduced to Japan as a famous spice during Tang Dynasty (AD 710 to 784), and later entered Europe and then America in the 17th century (Li, 1993). Due to its ornamental, medical and nutritional value, CM appreciation had created an unique chrysanthemum culture in East Asia. The varieties of CM have increased from 300 in Qing Dynasty to over 1,000 nowadays (Zhang, 2008; Mao, 2020). The CM’s medical and nutritional values were recognized around 2,300 years ago (Chen, 2005; Zhang, 2008); in the Spring and Autumn Period CM firstly appeared in “*Li Sao*,” written by the great poet Yuan Qu, as an edible vegetable. Later it was recorded as a medicine in “*Shen Nong’s Classic of the Materia Medica*” in Han Dynasty. Presently, CM is one of the most commonly consumed food, herbal medicine and tea beverage in China and adjacent countries (Lai et al., 2007; Jiang et al., 2013). CM is extensively used by healthcare providers to treat conditions such as dizziness, photophobia with lacrimation, conjunctivitis, headache with fever, red eyes, swollen poison and boils, among others (Chinese Pharmacopoeia Commission, 2020).

Modern studies found that *Chrysanthemum* and related genera contain significant amounts of volatile oils, flavonoids and hydroxycinnamoyl-quinic acids (Beninger et al., 2004; Jiang et al., 2004; Chen et al., 2007; Clifford et al., 2007; Lai et al., 2007; Wang et al., 2008a,b; Duan et al., 2022), and have extensive biological activities (Figure 1), e.g., anti-inflammation, antioxidation, vasodilation, protecting cardiovascular system, anticancer, inhibiting aldose reductase and anti-mutagenic (Zhang et al., 2000; Rajic et al., 2001; Peng et al., 2006; Shao et al., 2020). Given the long use history and important role of *Chrysanthemum* taxa, and in order to better preserve and utilize *Chrysanthemum* resources, a comprehensive review is necessary to synthesize information from existing studies of their phylogeny, biodiversity, chemodiversity and chemotaxonomy. In this study, the up-to-date information of *Chrysanthemum* were collected from major databases including NCBI PubMed, Google Scholar, Web of Science, SciFinder, Wiley online, Elsevier ScienceDirect and China National Knowledge Infrastructure (CNKI), using the keywords such as “Chrysanthemum,” “juhua,” “phylogenetic,” “biosynthesis,” the respective phytometabolite name, the respective genus/species name, and the like. Additionally, materia medica books and patents have made their contribution to the summary of botany, traditional uses and cultural significance of *C. morifolium*. The phylogenetic kinship, origin of genuine varieties, and the chemical ingredients and their bioactive effects of *Chrysanthemum* taxa were elaborated from the perspective of pharmacophylogeny, so as to facilitate a holistic understanding of *Chrysanthemum* and related genera and promote the sustainable conservation and utilization of relevant natural resources.

## Biodiversity and phylogenetic relationship

### Intergeneric relationship within Asteraceae tribe Anthemideae

The family Asteraceae, also called Compositae, consists of over 32,000 known species of flowering plants in over 1,900 genera within the order Asterales (Mandel et al., 2019). There are more than 200 genera and more than 2,000 Asteraceae species in China, which are distributed all over the country. Anthemideae is a tribe of the subfamily Asteroideae, plants of which are distributed worldwide with concentrations in central Asia, Mediterranean Basin, and southern Africa. Most species of plant known as chamomile belong to genera of this tribe. There are about 1,800 species classified in 111 Anthemideae genera (Oberprieler et al., 2007). This tribe is divided into 14 subtribes, and the genus *Chrysanthemum* belongs to the subtribe Artemisiinae (Figure 1).

Artemisiinae has 19 genera, 15 of which are distributed in East Asia, and there are eight endemic genera. This subtribe is the most important Anthemideae subtribe in East Asia, including more than 80% of the Anthemideae genera. Based on the extensive collection of East Asian Anthemideae resources, the systematic evolution was studied by means of pollen morphology, molecular systematics, molecular markers and so on (Zhao, 2007). The intergeneric crossing between cultivated *C. × grandiflorum* and *Ajania pacifica* was conducted, as well as self-crosses and backcrosses of hybrid F1. The genetic performance and cytological behavior of traits in different hybrid combinations and generations were probed to elucidate the relationship between parental genomes and their roles in different generations of hybrids. The East Asian Anthemideae taxa are mainly diploid and tetraploid, and many monotypic genera native to East and Central Asia, e.g., *Opisthopappus* Shih and *Crossostephium* Less., etc., are diploid. It is speculated that the ancestor of the tribe should be diploidy. Within the genus *Ajania* Polj., the chromosome ploidy differs between species and intraspecific populations.

East Asian Anthemideae can be divided into three groups according to the outer wall decoration of pollen (Zhao, 2007). The first group has obvious thorn-like decoration on the surface, the thorns are larger, longer than 1.5 μm, the base is expanded, and there are hole-like perforations from middle of the thorn to the thorn base. This group includes genera *Glebionis* and *Argyranthemum* of the subtribe Chrysanthemidae, *Hippolytia*, *Opisthopappus*, *Pyrethrum* and *Tanacetum* of subtribe Tanacetinae, *Leucanthemum* and *Nipponanthemum* of subtribe Leucanthemidae, *Matricaria* of subtribe Matricariinae, *Achillea* of Achilleinae, *Chrysanthemum*, *Ajania* and *Brachanthemum* of subtribe Artemisiinae. The second group has degenerate small spines or inconspicuous spines, basically less than 0.5 μm. This group includes genera *Artemisia*, *Seriphidium*, *Crossostephium*, *Elachanthemum*, *Neopallasia*, *Ajaniopsis*, *Filifolium* and *Kaschgaria*. The third group has shorter surface thorn-like ornamentation, which is, however, very obvious, and its length is between the first and second groups, 1.0–1.5 μm; it is conical, the base does not descend, and the apex is pointed. This group includes genus *Stilpnotepis*, *A. salicifolia* and *A. variifolia* of *Ajania*. Based on the outer wall decoration of pollen, *Phaeostigma* is proposed to be a separate genus from *Ajania*.

**Figure 2:**
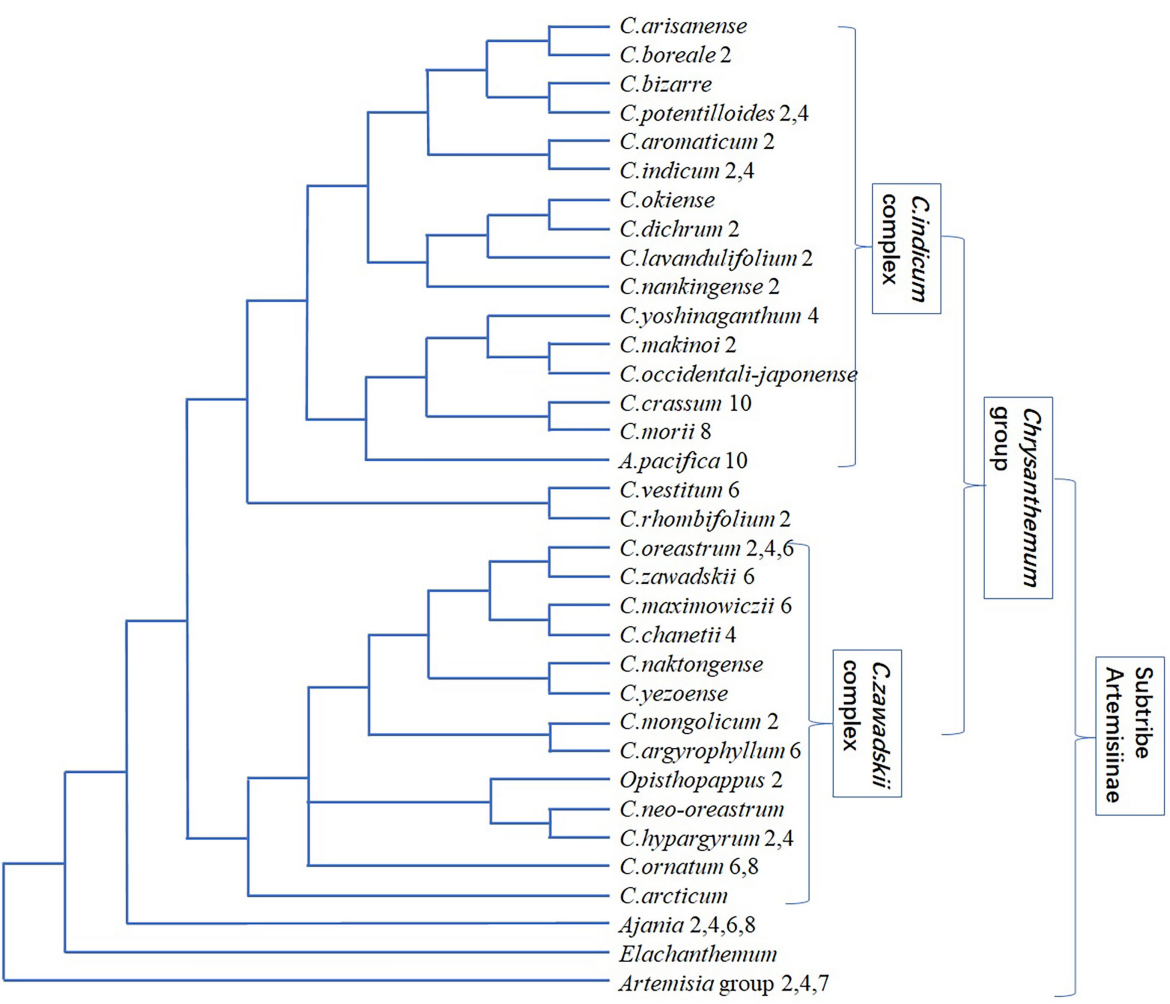
Phylogenetic relationship of *Chrysanthemum* taxa. The number after the taxon name represents the ploidy level. This schematic is compiled mainly based on Shen et al., 2021, as the phylogenetic topology inferred from low-copy nuclear genes and nrITS could be more convincing than those inferred from cp sequences (Liu et al., 2012; Lu et al., 2022a). It should be noted that it is challenging to distinguish taxa from each other within *C. indicum* complex and *C. zawadskii* complex, respectively.

To better understand the intergeneric relationships and taxonomic position of small Asian genera of Anthemideae, the sequences of nuclear ribosomal internal transcribed spacer (nr ITS) and chloroplast (cp) trnL-F intergenic spacer (IGS) were used to infer the phylogenetic relationship of 48 Anthemideae taxa (Zhao et al., 2010). The trnL-F sequence was of poor resolving power, but some deletions and insertions were useful in interspecific and intergeneric circumscriptions. Both ITS and ITS/IGS phylogenies suggest two major clades. The clade A is subtribe Artemisiinae, consisting of two groups; one group includes *Chrysanthemum*, *Arctanthemum*, *Ajania*, *Opisthopappus* and *Elachanthemum*, while the other includes *Artemisia*, *Crossostephium*, *Neopallasia* and *Sphaeromeria*. Within the former group, *Chrysanthemum*, *Arctanthemum* and *Ajania* were closely related to each other, and the delimitation of genera *Chrysanthemum* and *Ajania* was ambiguous. The successful hybridization between *C. × grandiflorum* and *A. pacifica* and the normal meiotic chromosome behavior of the hybrid also indicate the close relatedness between two genera (Zhao, 2007). *Phaeostigma* was not in *Chrysanthemum* group, which was confirmed by the 6-bp insertion in trnL-F. *Brachanthemum* was excluded, and *Elachanthemum* was included in this group. *Opisthopappus* of subtribe Tanacetinae was proposed to be in the subtribe Artemisiinae, *Chrysanthemum* group (Hong et al., 2015; Figure 2). The molecular phylogeny facilitates the inference of evolution of pollen and capitulum characters. Disentangling old hybridization events may be compromised by the set of evolutionary processes accumulated subsequently (Criado Ruiz et al., 2021), particularly in areas with past climatic instability. The high-throughput sequencing data will facilitate understanding the role and impact of reticulate evolution in the phylogenetic puzzle.

In an amplified fragment length polymorphism (AFLP) analysis of Artemisiinae, 1705 polymorphic bands were obtained (Zhao, 2007). According to the similarity coefficient, both within the whole subtribe, its related groups, and within *Chrysanthemum* and *Ajania*, the high genetic diversity was revealed, indicating obvious differentiation between genera and species. In the cluster diagram, *Elachanthemum* and *Neopallasia* are at the base of the whole subtribe, while *Brachanthemum* is at the base of *Chrysanthemum*-*Ajania* branch. The population of *Ajania* is always at the base of *Chrysanthemum* population, and the latter may have evolved from different species or populations of *Ajania* in parallel. The close relatedness between *Opisthopappus* and *Chrysanthemum*-*Ajania* was also confirmed by AFLP results (Zhao et al., 2010). Based on the results of cytology, pollen morphology, molecular sequence analysis and AFLP, it is believed that the East Asian Anthemideae could originate in the Eurasian continent, and the ancestral species of Eurasian continent spread eastward and gradually evolved throughout Central Asia and East Asia. Some genera distributed in Central Asia, e.g., *Kaschgaria*, may be the relatively primitive groups of Artemisiinae.

**Figure 3:**
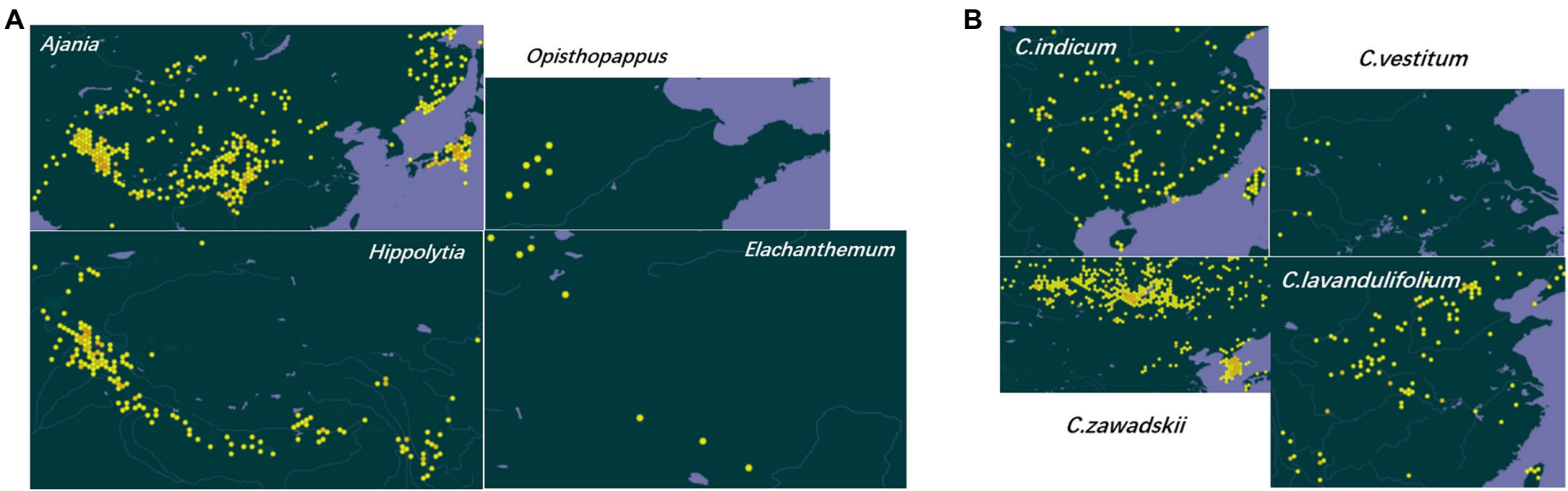
Geographical distribution of representative taxa of Asteraceae subtribe Artemisiinae. **(A)** Genus *Ajania* is produced in the vast areas of China except the southeast, Mongolia, North Korea, northern Afghanistan and Russia; *Opisthopappus* is endemic to Taihang Mountains, China; *Elachanthemum* is distributed in northern China, northwestern China and Mongolia; *Hippolytia* is produced in central Asia and the Himalayas. **(B)** *C. indicum* is produced in Northeast China, North China, Central China, South China, Southwest China, India, Japan, North Korea and Russia; *C. vestitum* is produced in western Henan, western Hubei and western Anhui, China; *C. zawadskii* is produced in Heilongjiang, Jilin, Liaoning, Hebei, Shanxi, Inner Mongolia, Shaanxi, Gansu and Anhui of China, Mongolia, Russia and Europe; *C. lavandulifolium* is produced in Jilin, Liaoning, Hebei, Shandong, Shanxi, Shaanxi, Gansu, Qinghai, Xinjiang (eastern), Jiangxi, Jiangsu, Zhejiang, Sichuan, Hubei and Yunnan of China, Korea and Japan. The geographic distribution data are retrieved from Global Biodiversity Information Facility (https://www.gbif.org/).

The geography and ecology play a key role in driving species diversification of Anthemideae and in shaping genotype/phenotype of species. The multi-phased orogenesis of Qinghai-Tibetan Plateau (QTP) and global climate changes over late-Miocene has profoundly influenced the environments and evolution of Anthemideae plants in QTP and adjacent regions (Shen et al., 2021). The DNA sequences of seven low-copy nuclear genes and nrITS were combined to reconstruct a time-calibrated phylogeny of subtribe Artemisiinae (Figure 2). In the monophyletic *Chrysanthemum* group, *Chrysanthemum* and *Ajania* were well resolved, agreeing with capitulum morphology and suggesting the low resolving power of cp and ITS markers (Zhao et al., 2010; Hong et al., 2015; Tyagi et al., 2020; Masuda et al., 2022). Within *Chrysanthemum*, the *C. indicum* complex and *C. zawadskii* complex diverged, which temporally coincide with the late Cenozoic uplift of Northern QTP and associated climatic heterogeneity between eastern and central Asia. The origin of *Chrysanthemum* group might be in Central Asia, then *Chrysanthemum* migrated eastward, in contrast to the *in situ* diversification of *Ajania* (Figure 3). The *C. indicum* complex and *C. zawadskii* complex have distinct distributions in East Asia, i.e., the former in more southern and the latter in more northern regions. The distribution patterns are related with the niche differentiation of different lineages and environmental heterogenization.

Through distant hybridization and aggregating germplasms, the wild Anthemideae genetic resources with excellent resistance, e.g., cold resistance, drought resistance, waterlogging resistance, etc., can be used in modern chrysanthemum breeding (Sun et al., 2010a; Hong, 2014) to realize the innovation of germplasm resources. While breeding new varieties, some basic data for exploring the genetic relationship between *Chrysanthemum* and related genera can be obtained (Wu, 2014), and the phylogenetic positions of some ambiguous taxa can also be clarified. With conventional hybridization methods or embryo rescue conditions, distant hybrids were obtained between *Chrysanthemum* and genera such as *Ajania*, *Crossostephium* (subtribe Artemisiinae), *Opisthopappus*, *Tanacetum*, *Pyrethrum* (subtribe Tanacetinae), *Argyranthemum*, *Glebionis* (subtribe Chrysanthemidae), *Leucanthemella* (subtribe Leucanthemidae; Zhao et al., 2008; Sun et al., 2010a; Shao, 2018). *Chrysanthemum*, *Ajania* and *Opisthopappus* are closely related (Figures 1, 2), and their hybridization is easier to be successful. *Brachanthemum* and *Hippolytia* are close to the clade of *Chrysanthemum* group, and their hybridization with *Chrysanthemum* (e.g., CM, *C. chanetii* and *C. naktongense*×*C. argyrophyllum*) can be successful (Hong, 2014; Xie, 2016). The hybrid without emasculation between *Hippolytia* and *Nipponanthemum* was obtained (Hong et al., 2015); on the ITS tree, both genera are closer to *Brachanthemum* than to *Chrysanthemum*. *Chrysanthemum* sensu lato includes around 38 genera, of which 27 are distributed in China (Shao, 2018). There are abundant wild resources and excellent stress resistance in *Chrysanthemum* sensu lato, which is of great significance to improve cultivated chrysanthemum breeding.

The chromosomal ploidy changes are abundant in *Chrysanthemum* (Figure 2), from diploid to decaploid, and there are also ploidy differences in different populations within the species. *C. crassum* (2n = 10x =90), *C. indicum* (4x = 36) and *C. nakingense* (2x = 18), as the female parent, respectively, were crossed with *Ajania myriantha* (2x = 18), *Crossostephium Chinense* (2x = 18), *Opisthopappus taihangensis* (2x = 18) and *Tanacetum vulgare* (2x = 18), respectively, to explore the genetic relationship between *Chrysanthemum* and its Anthemideae relatives (Tang, 2009). The F1 progeny of *C. indicum*×*Crossostephium Chinense* grew healthily and bloomed luxuriantly; the F1 progeny of *C. indicum*×*O. taihangensis* grew well, but there was no flower bud differentiation. The F1 progeny of *C. indicum*×*T. vulgare* did not grow normally in the field. It is speculated that the genetic relationship between *Chrysanthemum* and related genera from close to far is *Ajania* (Wu, 2007), *Crossostephium*, *Opisthopappus* and *Tanacetum*. In genomic two-color fluorescence *in situ* hybridization (FISH), the chromosomes of *Crossostephium* and *Tanacetum* were not observed in F1 progenies of CM cultivar “Tianzhuiyulu (falling jade dew)” × (*C. indicum*×*Crossosstephium Chinense*) and “Tianzhuiyulu” × (*C. nakingense*×*T. vulgare*), indicating that the chromosomes of two related genera may have been excluded. However, in F1 progeny of “Tianzhuiyulu” × (*C. crassum*×*Crossostephium Chinense*) the genomic chromosomes of three parents were observed, which realized the innovation of CM germplasm and enable full use of the excellent germplasm of *Chrysanthemum* related genera to improve CM varieties.

### Intrageneric relationship of *Chrysanthemum* and origin of CM

The genus *Chrysanthemum*, associated with polyploidy and hybridization, undergoes rapid speciation and has about 40 species, most of which are distributed in East Asia (Liu et al., 2012). Many taxa are narrowly distributed and grow in specific habitats. The *Chrysanthemum* taxa vary greatly in morphology and ploidy (Figure 2), and the interspecific relationships are not fully understood. The genome size values of 15 species fall into three groups (Luo et al., 2017), which positively correlate with three ploidy levels (2×, 4×, 6×), and there was a genome downsizing after polyploidization in *Chrysanthemum*. Two major phylogenetic clades were inferred based on ITS and trnL-F sequences, i.e., *C. indicum* complex and *C. zawadskii* + *Ajania* group. The genome size and 1Cx values (DNA content of one non-replicated monoploid genome with chromosome number x) in the former group were significantly lower than those in the latter group, even though both have same ploidy level, and they were also correlated with latitude and morphology. The 1Cx values of diploid *Chrysanthemum* species were similar to those of *C. indicum* (Mt. Tianzhu) and *C. indicum* (Fujian) indicate that some populations of tetraploid *C. indicum* originated by autoploidy (Li et al., 2013). The genome size of *C. chanetii* is significantly larger than that of other tetraploids, while *C. vestitum* is smaller than other hexaploids, but the relationship between genome size and evolutionary time cannot be determined due to limited sampling. The divergence between *Chrysanthemum* and *Ajania* may be relatively recent, presumably temporally similar to the divergence of *C. zawadskii*. Hybridization and gene flow between taxa occurred frequently during the evolutionary history of *Chrysanthemum* and *Ajania*, causing considerable incongruency of cp and ITS trees (Li et al., 2014; Lu et al., 2022a). The gene flow had occurred between *C. indicum*, *C. nankingense* and *C. lavandulifolium* (Yang et al., 2006; Liu et al., 2012), and between *Cheiracanthium mongolicum* and *C. chanetii* (Luo et al., 2017), which could be involved in the origin of polyploids of *C. indicum* and *C. zawadskii* complex, respectively.

The ITS and cp rpl16 sequences were combined to resolve the interspecific relationship of *Chrysanthemum* (Wu, 2007). *C. indicum* and *C. nankingense* (once regarded as a variety of the former), *C. dichrum* and *C. lavandulifolium*, *C. japonicum* and *C. yoshinaganthum*, *C. crassum* and *C. rhombifolium* are closely related. The meiotic behavior of several *Chrysanthemum* taxa and their hybrids suggested that three diploid species are closely related to each other (Cui, 2004), among which *C. dichrum* and *C. lavandulifolium* are the closest (Figure 2), followed by *C. lavandulifolium* and *C. nankingense*, and *C. nankingense* and *C. dichrum* are the furthest. *C. japonense* and *C. vestitum*, *C. lavandulifolium* and *C. boreale*, *C. zawadskii* and *C. weyrichii* are also closely related. However, they are still independent species and should not be combined into the single species. In neighbor-joining analysis of DNA sequences, *C. japonicum*, *C. arcticum*, *C. japonense*, *C. vestitum* and their respective subspecies/variety got together in pairs, showing a close kinship. The small genetic distance between *C. zawadskii* and *C. zawadskii* var. *latilobum* shows their close genetic relationship. However, in the morphological cluster analysis, these species and their variety/subspecies were not well aggregated, indicating the inconsistency between the phenotypic evolution and genetic evolution of *Chrysanthemum*, with large differentiation of morphological traits and small genetic differentiation of DNA sequences.

In the clustering analysis based on 24 morphological characters and one biological character, Chinese *C. indicum*, *C. lavandulifolium*, *C. nankingense* and *C. dichrum* clustered together (Wu, 2007), and most *Chrysanthemum* taxa originating in Japan clustered together, indicating that there is an obvious geographical isolation between Chinese and Japanese *Chrysanthemum* plants. The Japanese wild species evolutionarily appeared later than Chinese ones. In PCR-RFLP, three CM varieties “Yinxing,” “Jinlingwanxia” and “Shenma” had the same enzyme digestion pattern as that of wild *Chrysanthemum* in China, indicating their close genetic relationship. These CM cultivars have close genetic relationship with *C. indicum*, *C. vestitum* and *C. nankingense*, but they have the closest relationship with *C. chanetii*, and are far from *C. ornatum* and *C. japonense*, although wild species native to Japan have also played a positive role in enriching modern CM varieties. According to the morphological characteristics and molecular phylogeny, the wild *Chrysanthemum* species were classified into four groups, the indicum group, makinoi group, zawadskii group, and *Ajania* group (Nakano et al., 2021).

The multiple differentiation and hybridization/polyploidization cycles characterize the reticulate evolution of *C. indicum* complex (Yang et al., 2006) and *C. zawadskii* complex (Lu et al., 2022a), causing the difficulties in systematic classification. The DNA sequences of single-copy nuclear CDS (chrysanthemyl diphosphate synthase) gene and seven cpDNA loci were used to infer the phylogenetic relationship of 32 *Chrysanthemum* taxa and 11 species of the allied genera (Liu et al., 2012). It was found that the affinity between *Chrysanthemum* and *Ajania* is very close, and the resolving power of CDS and cpDNA markers were not enough. When eight nuclear sequences were used (Shen et al., 2021), *Chrysanthemum* and *Ajania* were well separated with only a few exceptions (Figure 2), which agrees with traditional taxonomy mainly based on capitulum morphology. In *Chrysanthemum*, two Chinese endemic species with white ray flowers, *C. rhombifolium* and *C. vestitum*, are sister to *C. indicum* complex with yellow/white ray flowers (Figure 2). The *C. indicum* complex has a and b branches; species of ‘b’ have island distributions, and species of ‘a’ are mostly distributed in mainland China. The *C. zawadskii* complex (Figure 2) has white to purple ray flowers with continuous color variation, and *Opisthopappus* falls into this clade. A Hyb-Seq based phylogenetic study found that *C. zawadskii*, *C. naktongense*, *C. chanetii* and *C. maximowiczii* intermingled in a clade with highly complicated phylogenetic relationships (Lu et al., 2022a), and the leaf morphology and phylogenetic analyses were difficult to distinguish them from each other, arguing that they have not yet differentiated into independent species and supporting the taxonomic treatments of *C. zawadskii* complex.

When nuclear CDS was used to infer the phylogenetic relationship, *Phaeostigma* is more closely related to *Chrysanthemum* + *Ajania* than other genera (Liu et al., 2012). According to pollen morphology and DNA data, *A. purpurea* is a member of *Phaeostigma*. The species differentiation in *Chrysanthemum* could be correlated with geographic and environmental factors. The *C. zawadskii* complex is distributed in northern China and *C. indicum* complex in southern China (Figure 3). Many polyploid species, e.g., *C. argyrophyllum*, could originate from divergent ancestors via allopolyploidization. The geographic/ecological conditions, hybridization and polyploidy play important roles in the divergence and speciation of *Chrysanthemum*. The comparative transcriptome analysis of CM and diploid *C. boreale* revealed whole-genome duplication (WGD) and gene selection patterns in these taxa (Won et al., 2017); the transcriptomes of *C. rhombifolium* and *C. dichrum* were also preliminarily characterized (Liu et al., 2021a; Zhang et al., 2021a), but the phylotranscriptomic analysis covering more *Chrysanthemum* species has not been reported.

In order to clarify the origin of CM, the investigation and collection of Chinese medicinal *Chrysanthemum* varieties with less germplasm erosion, ancient ornamental big CM (Daju in Chinese) varieties, *Chrysanthemum* sensu stricto and related genera were highlighted (Zhou, 2009); these plants were used as the materials of various experiments for the origin of CM by using different technical methods. Natural hybridization and cultivation play an important role in the origin of CM. In the distant hybridization of four years, 47 combinations were carried out, and 3,225 seeds, 675 hybrid seedlings and 30 flowering variant seedlings were obtained. *C. vestitum* and *C. indicum*, *C. vestitum* and *C. zawadskii*, *C. vestitum* and *C. lavandulifolium* were easy to cross successfully, and seeds were obtained. In the same distribution area of *C. vestitum* and *C. indicum*, the flowering period of the former is slightly earlier than that of latter, and they meet in flowering period (Wang, 2020). The F1 generation had great variation, and the inflorescence, flower color and leaf type were close to modern CM. The F2 progeny showed greater variation after natural pollination, which was very similar to ornamental small CM (Xiaoju). Hybridization is the main approach of origin of CM. *C. vestitum*, *C. indicum*, *C. zawadskii*, *C. lavandulifolium* and *C. dichrum* are closely related and play a role in the origin of CM to varying degrees. There are more frequent natural crosses between *C. indicum* and *C. vestitum*, possibly due to the weak reproductive isolation between tetraploid and hexaploid (Wang, 2020). The phylogenetic analysis of nuclear LFY sequences suggested that different cultivars had different ancestors (Ma et al., 2016). *C. indicum*, *C. zawadskii* and *C. nankingense* might be the direct ancestors of most CM cultivars examined. *C vestitum* and *C. lavandulifolium* might be the ancestor of some cultivars. Another line of support is from simple sequence repeat (SSR) markers mined from expressed sequence tags (ESTs; Fan et al., 2019) of CM. Transferability of EST-SSR primers among *Chrysanthemum* species was identified in *C. indicum* (96–100%), *C. nankingense* (100%), *C. chanetii* (96%), *C. zawadskii* (96%), and *C. indicum* var. *aromaticum* (92%).

The cluster analysis of ISSR (inter-simple sequence repeat)-PCR showed that the genetic relationship between *C. vestitum* and Daju/medicinal CM is the closest (Zhou, 2009), followed by *C. vestitum* of Yichang, Hubei Province (*C. vestitum var. vestitum*, Wang, 2020), *C. zawadskii* and *C. nankingense*. The main evolution mode of CM could be: Wild *Chrysanthemum* → medicinal CM (economic original chrysanthemum) → ornamental CM. Based on the research results on the origin of CM for half a century, the macro-morphology is combined with cytology, palynology, isozyme, DNA molecular markers, which are further combined with artificial hybridization, field resource investigation, introduction and domestication, and historical analysis, and it is concluded that the cultivated CM (original CM beginning in the era of poet Tao Yuanming, Jin Dynasty) originated in China; it is a hybrid cultigen complex, and is mainly produced by natural interspecific hybridization of some wild species in the middle reaches of Yangtze River (Hubei, Anhui and Henan). After long-term artificial repeated selection of some special variation types, it is carefully cultivated. The main parents are *C. vestitum* and *C. indicum*. Later, *C. zawadskii*, *C. lavandulifolium*, *C. indicum var. aromaticum*, *C. nankingense* and *C. dichrum* participate in the evolution of CM through pollen to varying degrees; the germplasm introgression enhanced the diversification of CM genes, so today’s ever-changing CM is formed. The recurrent hybridizations between several wild progenitor species contributed to the evolutionary novelty of CM (Song et al., 2018), and cultivation/selection also play a significant role in the formation of CM.

Many wild *Chrysanthemum* species may be involved in the complex formation process of CM. The ISSR-based UPGMA (unweighted pair group method with arithmetic means) clustering suggested that the *Chrysanthemum* species evolved from low ploidy to high ploidy (Liu, 2010); it can generally be inferred that the flat petal is the basic petal shape of ornamental CM. *C. nankingense* and CM had the closest genetic relationship, *C. chanetii*, *C. japonicum*, *C. japonicum var. wakasaense* were also closely related to CM, and *C. indicum var. aromaticum* was farthest related to other samples. The random amplified polymorphic DNA (RAPD) analysis showed that the genetic diversity of wild species was more abundant than that of CM. The cultivated Xiaoju, rather than Daju, was genetically closer to the wild *Chrysanthemum* species, and the petal shape and flower diameter can be used in the classification of CM. AFLP markers were used to detect the relationships among 12 wild accessions and 62 groundcover cultivars (Chen et al., 2013). The genetic variation is abundant in chrysanthemums. The 74 accessions were classified by UPGMA; the genetic relationship was the most relevant factor in AFLP-marker clustering, and petal type was also informative. AFLP technology could be very efficient for discriminating *Chrysanthemum* species and related genera and reconstructing their genetic relatedness.

### Intraspecific relationship of CM and origin of cultivars

Several genus names have been used for CM, such as *Matricaria, Anthemis, Pyrethrum, Chrysanthemum* and *Dendranthema*, reflecting its ambiguous phylogenetic position. The species names of *grandiflora* vs. *morifolium* were contentious. The International Code of Botanical Nomenclature changed the defining species of genus to *C. indicum* (Figure 1), and restored the florist’s chrysanthemum to the genus *Chrysanthemum* (Poe and Swofford, 1999). CM is a perennial herb with 50–140 cm height, has erect and slightly purplish red stems (FRPS, 1983); the base of the plant is woody, with much branching covered by densely white pubescent. The leaves of CM have a shape of ovate to lanceolate, with a color of dark green above and greenish beneath covered by densely white short hair on both sides. The flower heads are on the branches separately or aggregated with each other, with 2.5–15 cm in diameter; the involucral bracts of CM have multiple layers and ligulate flowers have diverse colors. The tubular flowers are yellow, blossoming in September to October in the northern hemisphere.

CM has been cultivated for more than 1,600 years and is one of the top ten traditional famous flowers in China. It has rich variation, strong adaptability, wide distribution and complex genetic background, which has brought great difficulties to the investigation and protection of germplasm resources, identification and classification, genetic diversity analysis, and the like. The genetic diversity of 53 CM germplasm samples with different flower diameter, petal type and flower color type was studied using conserved DNA-derived polymorphism (CDDP) markers (Li, 2014) to provide a basis for the classification and identification of chrysanthemum varieties. In the cluster analysis of CDDP amplification results, 53 chrysanthemum materials were divided into six groups. Group 1 includes 30 Daju varieties and two Xiaoju varieties, group 2 contains 16 Xiaoju varieties, and group 3 includes two Xiaoju varieties. *C. nakingense*, *C. indicum* and *C. vestitum* were in groups 4, 5 and 6, respectively. The CM varieties were basically clustered according to flower diameter, which has no direct correlation with petal type and flower color. The phylogenetic analysis based on large sets of single nucleotide polymorphisms (SNPs) revealed that the Xiaoju types and potted/ground chrysanthemum, instead of Daju, are more closely related to the wild progenitor species (Chong et al., 2016).

**Figure 4:**
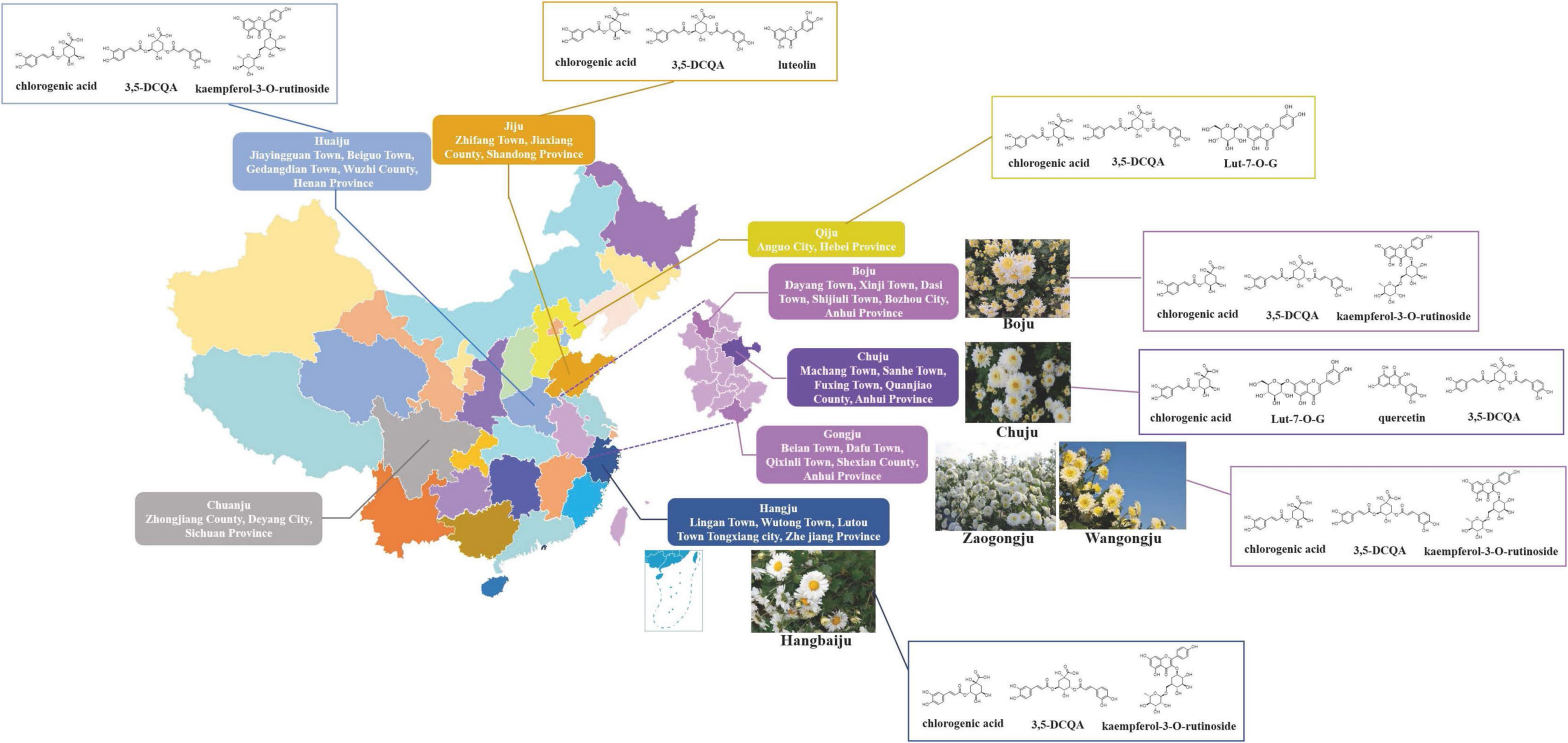
Geographical distribution, chemical and morphological characteristics of representative CM cultivars. The medicinal quality (Q) markers of each cultivar are shown: Boju, Huaiju, Gongju and Hangju: chlorogenic acid, 3,5-DCQA, and kaempferol-3-O-rutinoside (Lu et al., 2022b); Chuju: chlorogenic acid, Lut-7-O-G, quercetin, 3,5-DCQA (Yang et al., 2018b); Qiju: chlorogenic acid, 3,5-DCQA, and Lut-7-O-G (Peng et al., 2019); Jiju: chlorogenic acid, luteolin and 3,5-DCQA (Kang et al., 2022). The Q-marker of Chuanju of Zhongjiang, Sichuan Province is not reported. Morphology of medicinal CM: Boju: Inverted conical or cylindrical shape, sometimes slightly flattened and fan-shaped, 1.5–3 cm in diameter, discrete (Chinese Pharmacopoeia Commission, 2020). Involucral bracts dish-shaped; involucral bracts 3–4 layers, ovate or elliptic, herbaceous, yellow-green or brown-green, pubescent outside, margin membranous. Chuju: Irregular spherical or oblate spherical, diameter 1.5–2.5 cm. Ligulate flowers are white, irregularly twisted, involute, with shriveled edges, sometimes with light brown glandular dots; tubular flowers are mostly hidden. Gongju: Oblate spherical or irregular spherical, 1.5–2.5 cm in diameter. Ligulate flowers white or off-white, obliquely ascending, upper part reflexed, margin slightly involute and shriveled, usually without glandular dots; tubular flowers few, exposed. Hangju: It is dish-shaped or oblate spherical, with a diameter of 2.5–4 cm, and is often connected in several pieces. Ligulate flowers white or yellow, spreading or slightly folded, adhering to each other, usually without glandular dots; tubular flowers numerous, exposed. Huaiju: Irregular spherical or oblate spherical, diameter 1.5–2.5 cm. Ligulate flowers are the most, white or yellow, irregularly twisted, involute, with shriveled edges, and sometimes glandular dots can be seen; most of the tubular flowers are hidden.

The CM varieties are classified in terms of the parameters such as flowering stage, flower diameter, flower color, appearance of petals, cultivation form, leaf shape, among others. Both medicinal CM, i.e., *Flos Chrysanthemi* in TCM, and ornamental CM can be classified according to these external characteristics. Before Song Dynasty, the artificially cultivated CM was for food/medicine and viewing, and the wild relatives were also used as medicine. With the increased use of medical varieties, the cultivation flourished in Qing Dynasty, when CM’s genuine production areas were formed (Figure 4). The changes of CM producing areas in ancient times are shown in Supplementary Figure S1. With the passage of time, changes of cultivation sites and processing methods, eight famous CM categories are gradually formed (Figure 4); *Chinese Pharmacopoeia* contains five Daodi medicinal materials (geoherbs) of CM: Hangju, Boju, Gongju, Chuju, Huaiju (Chinese Pharmacopoeia Commission, 2020). There are also local varieties such as Qiju, Jinsi Huangju, Wuyuan Huangju and so on (Liu, 2020). Gongju and Hangju could be closely related (Wang et al., 1999, 2001), Huaiju, Jiju, Boju and Qiju are more closely related, while Chuju is an independent variety. Interestingly, among 26 antifungal terpenoids of 13 CM cultivars, longifolenaldehyde and β-selinene were identified only in Chuju (Xue et al., 2019). At present, Hang Baiju (Hangzhou white CM) and Gongju in the south of Yangtze River are mainly used for tea, while Huaiju, Boju, Chuju, Qiju and Jiju in the north of Yangtze River are mainly used for medicinal purposes.

Hangju, including Hang Huangju (Hangzhou yellow CM) and Hang Baiju, has been cultivated for about 400 years. Huangju was mainly used as medicine while Baiju was consumed as tea. In the 1950s and 1960s in Futianhe Town, Macheng, Hubei Province, Hangju (Big Baiju) was introduced from Tongxiang, Zhejiang Province (Chang et al., 2019). The large-scale planting began in the 1980s. The local CM is produced in Futianhe Town as the core production area, so they are also called “Macheng Fubaiju” or “Fubaiju.” The cultivars of Fubaiju mainly include Baiju and Jinju (golden CM), which can also be divided into two major varieties: “local early-flowering” and “local late-flowering” (Xiong, 2014); they were mainly used for tea. Surprisingly, the ISSR cluster analysis showed that the genetic distance between Fubaiju and Hang Baiju was relatively far (Xiong, 2014), and it was closer to heterophyllous chrysanthemum and Chuju in Chuzhou, Anhui Province.

Boju has been cultivated for at least 240 years, but its processing method of sulfur-fumigation arouses controversy. Twenty-one medicinal CM cultivars were heteroploidy (Wang et al., 2012), mostly hexaploid, and some were tetraploid (Huangyaoju) and pentaploid (Dahuangju). The karyotype of Da Boju, Xiao Boju, Chuju, “Xenogeneic Dabaiju” and “Little Baiju” is 2A, and the other 16 types are 2B. The chromosomes of 21 CM cultivars showed polymorphism, and the chromosome length, centromere position and satellite varied among cultivars. In a ISSR analysis (Lyu et al., 2008), both Da Boju and Xiao Boju were closer to different cultivars of Hangju. A cultivar-specific sequence-characterized amplified region (SCAR) marker was developed to detect Da Boju (Cai et al., 2022), which is morphologically similar to ornamental Xiaoju, and could be artificially evolved from Xiaoju.

Chuju, Hangju and Boju have deep notch correctitude-leaves (Li et al., 2010); unlike other cultivars, Chuju and Boju have no auricle. In a UPGMA analysis based on the DNA fingerprint patterns of SSR markers (Feng et al., 2016), Chuju was closer to Boju, followed by Gongju and Huaiju. In a ISSR analysis (Lyu et al., 2008), Chuju was closer to Jiju than to other medicinal CM. The uniqueness of Chuju was also validated by the quality evaluation and identification studies based on monoterpenoids and sesquiterpenoids (Wang et al., 2008b). Gongju is introduced for over 100 years mainly as tea beverage. Gongju grows in an environment of high altitude and high humidity during harvest, and the main processing method was drying by charcoal fire. In a ISSR analysis (Lyu et al., 2008), Gongju was closer to Hangju, agreeing with morphological and metabolomic investigations (Wang et al., 1999, 2001; Nie et al., 2019).

Huaiju is considered as the ancestor of medicinal CM because of its ancient records in medicinal uses. In a RAPD analysis (Dai et al., 2017), Huaiju was not clustered with Hangju, Gongju and *C. indicum* (*Chrysanthemi Indici Flos* (CIF) in TCM; Song et al., 2020), indicating the rich genetic diversity in chrysanthemum germplasm resources. Huaiju samples were clustered into a large group, indicating its high genetic purity. In a ISSR analysis (Lyu et al., 2008), *C. indicum*, *C. nankingense* and a hybrid between *C. indicum* and Gongju were grouped together. The 19 medicinal CM accessions were divided into two groups according to the origin. Most accessions originating in the north of Yangtze River had relatively close genetic relationships, while most accessions cultivated in the south also had relatively close relationships. The resolving power of ISSR is generally higher than that of RAPD (Xu et al., 2006; Luo et al., 2013), facilitating the clarification of inter-cultivar relationship of CM. Medicinal CM germplasm resources are indeed distinct at the molecular level; the differences among medicinal CM cultivation types are related to environmental factors (Huang et al., 2020), but to a greater extent are determined by their genetic factors. The quantitative analysis of morphological variation (Shao et al., 2011) and isozyme analysis (Ding et al., 2008) also help much in elucidating the inter-cultivar relationship, variety identification and conservation at different phenotypic levels.

## Phytometabolites and chemodiversity of *Chrysanthemum*

Plant pharmacophylogeny is an interdisciplinary subject that integrates plant systematics, phytochemistry, TCM, pharmacology and other multidisciplinary knowledge systems (Hao and Xiao, 2017, 2020), and plays an important role in guiding the conservation, development and utilization of medicinal plant resources. The core idea of plant pharmacophylogeny is that species in adjacent phylogenetic groups have relatively close genetic characteristics; the similar gene sequences lead to relatively close biosynthetic pathways of various phytometabolites in closely related taxa, manifesting a high degree of similarity in the chemical composition, i.e., (1) specific secondary metabolites are more likely to be distributed in multiple species that are genetically close and (2) natural products in closely related taxa have higher similarity in molecular skeleton composition and the relationship of derivation (Liu et al., 2021b). The similarity of the above two tiers of chemicals is manifested in the overall similarity of biological activity or therapeutic effect in clinical application. In practice, the concept of plant pharmacophylogeny has effectively guided the in-depth development and utilization of medicinal plant resources (Hao and Xiao, 2017, 2020), especially in the development of new medicinal plant species, so as to avoid the blindness in traditional research methods and guarantee the targeted research. Fully understanding the phytometabolites and chemodiversity of *Chrysanthemum* and related taxonomic groups is an indispensable basis for pharmacophylogenetic research.

### Volatile oils

**Figure 5:**
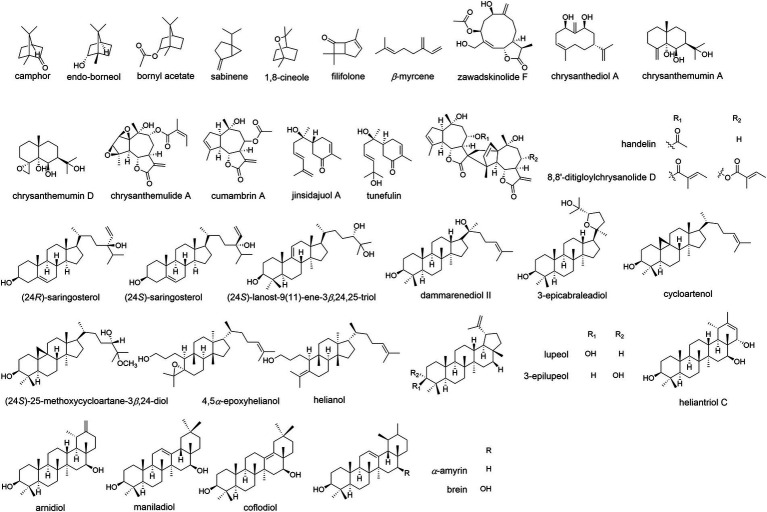
The molecular structure of representative terpenoid components of *Chrysanthemum.* A, Monoterpene: camphor, endo-borneol, bornyl acetate, sabinene,1,8-cineole, filifolone, β-myrcene. B, Sesquiterpene: germacrane-type: zawadskinolide F (anti-inflammatory, *C. zawadskii*), chrysanthediol A (anti-viral, CM); eudesmane-type: chrysanthemumin A (anti-viral, *C. indicum*), chrysanthemumin D (anti-viral, *C. indicum*); guaianolide-type: chrysanthemulide A (anti-tumor, anti-inflammatory, *C. indicum*), cumambrin A (treating osteoporosis, *C. ornatum, C. indicum, C. zawadskii*); bisabolene-type: jinsidajuol A (CM), tunefulin (*C. indicum*); others: handelin (anti-aging, *C. ornatum, C. indicum*), 8,8′-ditigloylchrysanolide D (anti-tumor, *C. indicum*). C, Triterpene: tetracyclic: stigmastanes: (24*R*)-saringosterol, (24*S*)-saringosterol (CM); lanostane: (24*S*)-lanost-9(11)-ene-3*β*,24,25-triol (CM); dammaranes: dammarenediol II (CM), 3-epicabraleadiol (anti-viral, CM); cycloartanes: cycloartenol (various bioactivities, CM), (24*S*)-25-methoxycycloartane-3*β*,24-diol (anti-inflammatory, CM); tirucallanes: 4,5*α*-epoxyhelianol (antitubercular, CM), helianol (anti-inflammatory, CM). pentacyclic: lupanes: lupeol (anti-inflammatory, *C. indicum*, CM), 3-epilupeol (antitubercular, CM); taraxeranes: heliantriol C (anti-tumor, CM), arnidiol (anti-tumor, CM); oleananes: maniladiol (antitubercular, CM), coflodiol (anti-tumor, CM); ursanes: *α*-amyrin, brein (CM).

Volatile oils were one of the most important bioactive components in Chrysanthemum species, which are mainly composed of hydrocarbons, terpenoids, alcohols, esters, ketones, aldehydes, among others (Ryu et al., 2019; Wang et al., 2021a). The herbivory-induced emission of volatile terpenes in Chrysanthemum is an indirect defense against pest by attracting natural enemies (Xu et al., 2021). The candidate terpene synthase (TPS) genes were identified by comparing the transcriptomes of healthy and pest-infested CM plants. Totally 46 terpenoids were identified in flower heads of 44 Chrysanthemum species/varieties and 43 were identified in the emission (Zhang et al., 2021b). The CM flowers had less terpenoids than their wild relatives, and displayed lower emission rates. The differences were principally determined by seven monoterpenes (camphor, endo-borneol, bornyl acetate, sabinene 1,8-cineole, filifolone, β-myrcene; Figure 5) and five sesquiterpenes (germacrene D, β-ylangene, (E)-β-farnesene, β-copaene, β-caryophyllene). At least 183 monoterpenoids have been reported in Chrysanthemum (Jiang et al., 2021b), e.g., the abundant borneol, camphor, *β*-pinene, *α*-thujone, and verbenone, etc. (Xie et al., 2012). CDS catalyzes the condensation of two molecules of dimethylallyl diphosphate to chrysanthemyl diphosphate, an irregular monoterpene (Liu et al., 2012). Irregular monoterpenes are mainly found in plants of the tribe Anthemideae. The monoterpene glycosides were identified from flowers of edible *C. “Kamiohno”* (Kurimoto et al., 2021).

In *C. indicum*, 17 monoterpenes and 27 sesquiterpenes were identified (Zhou et al., 2021). The recombinant CiTPS1 and 2 produced α-pinene, CiTPS3 was responsible for the production of sesquiterpenoids β-farnesene, petasitene, and α-bisabolene, while CiTPS4 contributed to the production of four monoterpenoids and three sesquiterpenoids. These functionally redundant genes were derived from WGD and segmental duplication of Chrysanthemum (Song et al., 2018; Nakano et al., 2021). Among the expanded C. nankingense gene families, the genes involved in the biosynthesis of monoterpenoids, sesquiterpenoids, triterpenoids are highly enriched (Song et al., 2018). The terpenoid synthase (TS) and cytochrome p450 (CYP) families are among the most highly enriched functional categories, and 219 TS genes (7 squalene synthases, 158 TPSs, and 54 triterpene cyclases) and 708 CYP genes were identified in C. nankingense genome, suggesting a significant expansion of TPS genes, explicitly in the TPS-a/−b subfamilies. Unlike Chrysanthemum, much less TS and CYP genes were identified in the other three Asteraceae genomes, i.e., artichoke, sunflower, and lettuce.

At least 207 sesquiterpenoids have been identified from Chrysanthemum (Chen et al., 2019; Jiang et al., 2021a,b), including 26 germacrane-type, 26 eudesmane-type, 64 guaianolide-type, 4 bisabolane-type, and 15 other-type sesquiterpenoids (Figure 5), with anti-inflammatory, antibacterial, anti-tumor, insecticidal, and anti-viral activities, etc. CM, *C. indicum*, *C. lavandulifolium*, *C. zawadskii*, and *C. ornatum* are rich in sesquiterpenes, especially the former two. Angeloylcumambrin B, cumambrin A and handelin were reported from more than one species. Germacrane-type sesquiterpenes are monocyclic, composing of a 10-member carbon ring, a methyl group at C-4 and C-10, and an isopropyl group at C-7. Eudesmane-type sesquiterpenes are bicyclic, consisting of two six-member carbon rings with methyl groups at C-4 and C-10, and an isopropyl group at C-7. Guaianolide-type sesquiterpenes have ternary rings consisting of a five-member ring, a seven-member ring, a five-member γ-lactone ring, and methyl groups at C-4 and C-10. They are more abundant than other types of sesquiterpene. The cyclization of farnesyl diphosphate to germacrene A could be the first committed step in sesquiterpene biosynthesis (Jiang et al., 2021b), and oxidations of (+)-germacrene A determine where additional cyclization occurs to generate guaianolides or eudesmanes. In GC/MS analysis of CM, *β*-humulene (*β*-caryophyllene) showed the highest contents, accounting for 16.3% of the total 58 detected volatiles (Sun et al., 2010b); ledene oxide-(I) was also abundant, amounting to 9.0% of total volatiles.

The constituent and yield of CM essential oils varied a lot depending on the harvest time, origin, processing methods, etc. (Xue et al., 2007; Yang et al., 2007; Wu et al., 2015). The flowers were blue-green in the early bloom stage and contained the most abundant volatile oil (Xue et al., 2007). There is correlation between floral volatile components and antioxidant activity of different CM cultivars (Yang et al., 2017a), therefore it is important to quantify contents of volatile oils in the respective cultivar. Chuju, Gongju and Qiju had 2.0–4.0 ml/kg volatile oil, Huangyaoju (a kind of Gongju), Boju and Huaiju had more (4.5 ml/kg), and Jiju had the highest volatile oil content of >10.0 ml/kg, whereas two kinds of Hangju had the lowest content of <1.0 ml/kg (Xu et al., 2005).

Based on the volatile chemical profile of flower tea, Hangju, Huangju, chamomile (*Matricaria chamomilla*), and a new CM “Xiaokuixiang” were well distinguished from each other (Wang et al., 2021a). The main volatile components in chamomile were the ester ethyl 2-methylbutanoate and alcohol 1,8-cineole monomer, but the content of ketones in chamomile was relatively low; ethyl 2-methylbutanoate and ketone 6-methyl-5-hepten-2-one were more abundant in “Xiaokuixiang.” The terpenoids 6-methyl-5-hepten-2-one and 1-menthol monomer were salient in Hangju and Huangju, but very few esters were detected in both, agreeing with their close phylogenetic relationship.

### Triterpenes and other terpenoids

More than fifty triterpenes were identified from *Chrysanthemum*, including tetracyclic and pentacyclic triterpenes (Figure 5). The former includes stigmastanes, lanostanes, dammaranes, cycloartanes (more than others), tirucallanes. The latter includes lupanes, taraxeranes (more than others), oleananes, and ursanes. Squalene, camphor, DL-α-tocopherol were among the top 10 volatile compounds of 12 γ-irradiated mutant cultivars of CM (Ryu et al., 2019), which are useful for classification and identification of chrysanthemum mutant cultivars. Though, the triterpene biosynthesis genes of *Chrysanthemum* and related taxonomic groups have not been reported, and the bioactivity of *Chrysanthemum* triterpenes is less studied. One diterpene (C20) was found in roots of 12 CM cultivars (Zhang et al., 2020).

### Flavonoids

**Figure 6:**
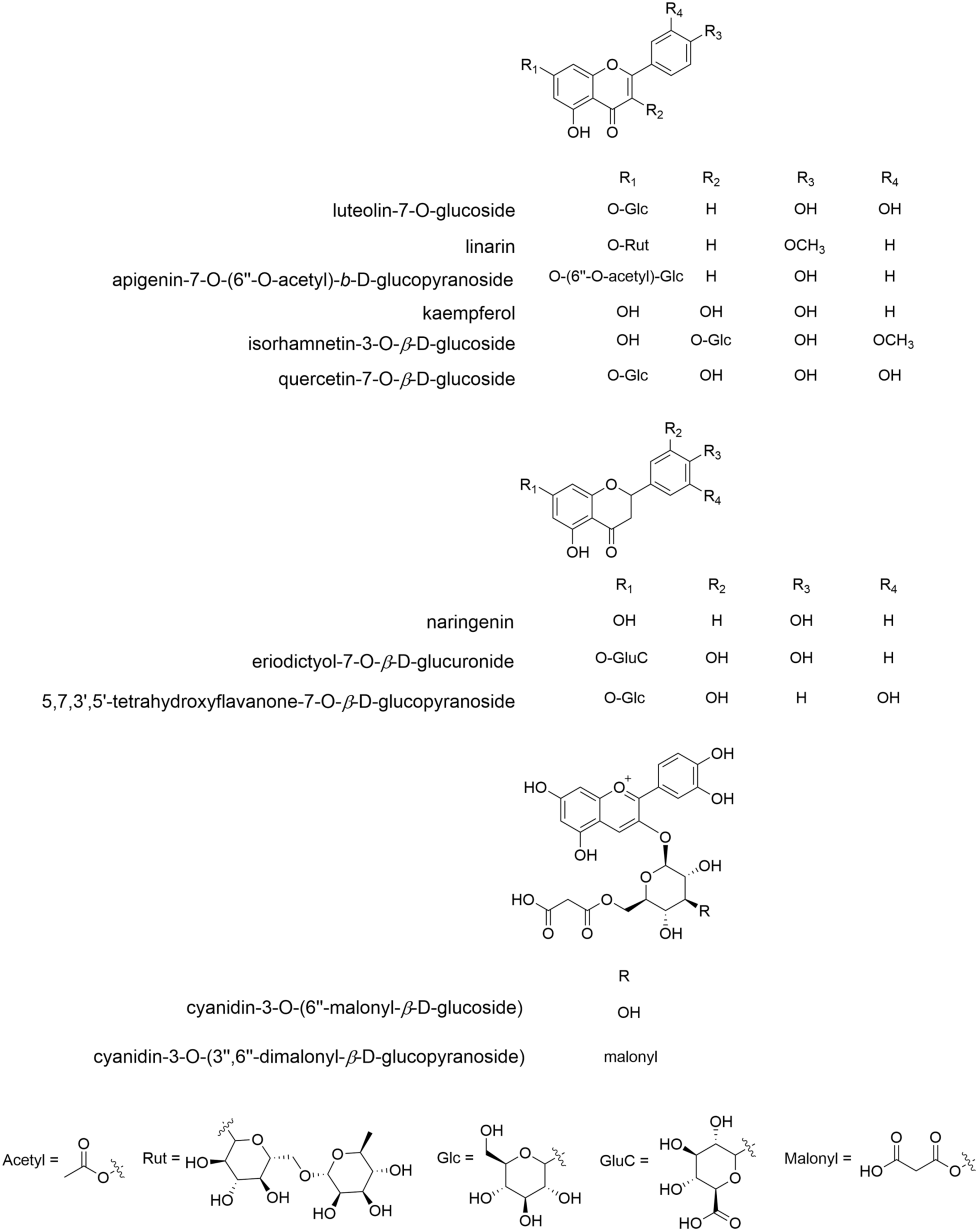
The molecular structure of representative flavonoid components of Chrysanthemum. Flavones: Lut-7-O-G (anti-inflammatory, antioxidant, relieving asthma, xanthine oxidase inhibitor, CM, *C. indicum*), linarin (antioxidant, anti-inflammatory, preventing acute lung injury, promoting osteogenic differentiation, inhibiting acetylcholinesterase activity, CM, *C. indicum*, C. zawadskii), Api-7-O-(6″-O-acetyl)-β-D-glucopyranoside (antioxidant, CM). Flavonols: kaempferol (antioxidant, anti-inflammatory, anticancer, CM, *C. indicum*), isorhamnetin 3-O-β-D-G (anti-inflammatory, CM, *C. indicum*), quercetin 7-O-β-D-G (various bioactivities, CM, *C. indicum*). C, flavonones: naringenin (treating depression, CM), eriodictyol-7-O-β-D-glucuronide (immunoregulation, C. zawadskii, *C. indicum*), 5,7,3″,5″-tetrahydroxyflavanone-7-O-β-D-glucopyranoside (*C. indicum*). D, anthocyanins: cyanidin-3-O-(6″-O-malonyl)glucoside (*C. grandiflorum*), cyanidin-3-O-(3″,6″-di-O-dimalonyl-β-glucopyranoside)(*C. grandiflorum*).

More than 78 CM flavonoids (Figure 6) are identified, and flavones are the most, followed by flavonols and flavonones. They fall into 12 categories (Jo et al., 2020; Mao, 2020), i.e., apigenin (Api) and derivatives (mainly glycosides), diosmetin (Dio) and derivatives, acacetin (Acn) and derivatives, kaempferol and derivatives, quercetin and derivatives, luteolin (Lut) and derivatives, hesperetin and derivatives, eriodictyol and their glycosides, isorhamnetin, baicalin, eupatilin, and anthocyanins. Among them, apigenin and derivatives are the most, mainly Api-7-O-6-AG (acetylglucoside), Api-7-O-G and the like, which is followed by luteolin and its derivatives, e.g., Lut-7-O-G (glucoside), Lut-7-O-MG (malonylglucoside) and so on. These are also abundant in *C. indicum* and *C. nankingense* (Zou et al., 2022). In *C. pacificum*, luteolin conjugates were mostly enriched in flowers (Farag et al., 2015), and non-flowering aboveground parts were rich in quercetin and methoxylated flavone conjugates. Root sample had the lowest contents of all flavones and dicaffeoylquinic acids (DCQAs).

Among 477 metabolites identified in *C. mongolicum* and *C. rhombifolium* (Duan et al., 2022), 69 are listed in the Chinese Pharmacopoeia (2015) and YaTCM database (Li et al., 2018). The flavonoids, e.g., linarin (11.64%), chrysanthin (5.03%), farnesin (3.03%) and genkwanin (2.75%), are abundant in *C. mongolicum*. The contents of genkwanin, trigonelline, Dio, narcissoside, 3,4-dihydroxyphenylacetic acid, linarin, N′,N′-p-coumarin, C-hexosyl-tricetin O-pentoside, chrysoeriol, Acn and kaempferol-3-O-gentiobioside were significantly different between *C. mongolicum* and *C. rhombifolium* (Duan et al., 2022), which could be biomarkers and inspire drug/food development.

Lut-7-G and quercitrin were the top two abundant compounds of CM, accounting for 85.7% of total flavonoids (Sun et al., 2010b). More Acn 7-O-galactoside resulted in lighter colored flowers and less Acn and kaempferol was associated with yellow flowers (Chen et al., 2021). The principal component analysis (PCA) of metabolic profile data separated 10 CM cultivars according to flower color rather than mutation rates (Sawada et al., 2019). Hot-H_2_O extraction of CM tea showed that most flavonoids and CQAs dissolved out at 30 min (Chen et al., 2021), with 20.977 and 8.958 mg/g gross weight (Learn how to make good CM tea).

The contents of taxifolin (dihydroquercetin) were clearly associated with the variation in mutation rates among 10 CM cultivars (Sawada et al., 2019). Taxifolin is a precursor of both cyanidin derivatives (pigments) and quercetin derivatives (colorless co-pigments modifying flower color). Higher accumulation of taxifolin could easily result in higher accumulation of cyanidin and/or quercetin derivatives by mutagenesis, causing changes in flower color. The anthocyanins cyanidin 3-G and cyanidin 3-(3′-malonoyl) glucoside were identified in 23 CM cultivars (Park et al., 2015), among which “Magic,” “Angel” and “Relance” had high amounts of anthocyanins and showed a wide range of red and purple colors in their petals. Dihydroflavanol 4-reductase (DFR) and anthocyanidin synthase (ANS) convert dihydrokaempferol to pelargonidin, a pink anthocyanidin. In CM, the B-ring of dihydrokaempferol is further hydroxylated to cyanidin by flavonoid 3′-hydroxylase (F3′H; Mekapogu et al., 2020). Due to the absence of flavonoid 3′5’-hydroxylase (F3′5′H), there is no delphinidin-based anthocyanin accumulated in CM petals. The accumulation of cyanidin and pelargonidin imparts pink to red-purple, orange to red, respectively. To date, the anthocyanin biosynthesis genes of wild *Chrysanthemum* taxa have not been reported; it is unknown whether F3′5′H is present in these taxa and how the variation of biosynthesis genes is involved in the contents of anthocyanins as well as spectrum of flower color.

The total flavonoid content of *Chrysanthemum* sensu lato varied between 4 and 10% (Shao, 2018), and its change was not obvious after the end of flowering. The content of linarin in CIF was significantly different among different habitats (Wei et al., 2021), where climatic factors, especially average annual precipitation, annual average sunshine hours, and annual average temperature, pointedly impacted on the linarin content. Some abundant flavonoids with content differences among CM cultivars can be used as indicators for quantitative identification (Mao, 2020), e.g., Lut-7-O-G, Api-7-O-G, Lut-7-O-β-G, Api-7-O-6-AG. The components with high content, large dispersion among cultivars, and large contribution to classification can be used as evaluation indicators for the quality of CM and cultivar identification, e.g., Api-7-O-6-AG, 3,5-DCQA, 4,5-DCQA, 3,4-DCQA, 3 -CQA. The content of some phytometabolites is relatively low, and their differences between CM cultivars are large, e.g., Api, Dio, 5-CQA, 4-CQA, Acn; some components were not detected in some CM cultivars, e.g., Api, Acn. These are not suitable as indicators for *Chrysanthemum* quality evaluation. The CM cultivar “XiaoYangJu” (a kind of Hangju) had the highest total flavone, total flavonol and total CQA, which is very useful in investigating the biosynthesis pathway of active ingredients and breeding cultivars with the highest specialized metabolite yield.

Whether the flavonoid/anthocyanin biosynthesis is conserved across *Chrysanthemum* species is actually unknown (Mekapogu et al., 2020). In general, the biosynthesis of flavonoids begins with the condensation of 4-coumaroyl-CoA and malonyl-CoA by chalcone synthase (CHS) and chalcone isomerase (CHI) to yield naringenin (Hao et al., 2015). In CM, the flavonoid 3-hydroxylase and F3′H are responsible for the synthesis of dihydrokaempferol and dihydroquercetin as the essential precursors for the corresponding flavonols. Naringenin could also be transformed into flavones via flavone synthase (FS) 1. Flavonols and flavones, via glycosyltransferase (GT), methyltransferase, hydroxyl transferase, and acyltransferase, are subject to diverse structural modifications to generate various flavonoids. In CM, the common feature of flavonol modification was 3-O-glycosylation (Chen et al., 2021), while flavones were involved in 7-O-glycosylation and 7-O-6′-acylation; the metabolic intermediates are then shunted into seven sub-pathways. There were significant positive correlations between flavonoids and corresponding flavonoid glycosides, and there were meaningfully positive correlations between Api and Aca, Api and Lut, Lut and Dio, indicating coordinated sub-pathways in CM, and possibly in its wild relatives. However, few data of other Artemisiinae species are available. The content of total flavonoids in different tissues of *O. taihangensis* was higher than that in corresponding tissues of *C. indicum* (Liu, 2013), but biosynthetic pathways have not been well investigated in these species. Seven *C. indicum* samples from different habitats were distinct in their flavonoid profile (Zou et al., 2022), which was also significantly different from *C. nankingense*. So far, a comprehensive metabolomic similarity analysis of as many *Chrysanthemum* taxa as possible is still lacking, and the role of genetic vs. climatic factors in metabolic differences of populations/species is elusive.

The enrichment of flavonoid biosynthesis genes in the *C. nankingense* genome was revealed by whole genome sequencing (Song et al., 2018). Two key genes, CHS and CHI, are significantly expanded in *C. nankingense* genome (17 CHS genes and eight CHI genes), which showed the differential expression among tissues, and there were significantly high expressions in flowers, suggesting the spatial and temporal regulation of flavonoid biosynthesis. Numerous flavonoid UDP-glucuronosyl and -glucosyltransferase (UGT) gene copies were also identified in *C. nankingense* genome, which is consistent with the phytochemical findings.

### Phenolic acids

**Figure 7:**
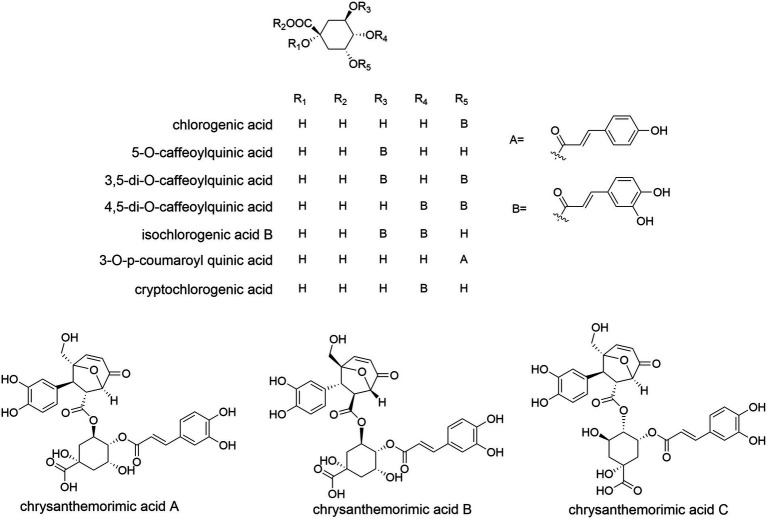
The molecular structure of representative phenolic components of *Chrysanthemum*. chlorogenic acid (=3-CQA), 5-CQA, 3,5-DCQA, 4,5-DCQA, isochlorogenic acid B, 3-O-p-coumaroyl quinic acid, cryptochlorogenic acid, chrysanthemorimic acid A/B/C.

Common phenolic acids of CM have long been known, e.g., chlorogenic acid, 4-CQA, 3,4-DCQA, 3,5-DCQA, 3,5-DC-epi-QA, 1,3-DC-epi-QA and ethyl caffeate (Xie et al., 2012; Yang et al., 2018a; Lu et al., 2022b). A rare 8-oxa-bicyclo[3.2.1]oct-3-en-2-one ring was reported (Yang et al., 2017b), which is formed through a [5 + 2] cycloaddition of CQA with a D-glucose derivative (Figure 7). *C. indicum* is rich in cryptochlorogenic acid and 3-O-p-coumaroyl quinic acid (Zou et al., 2022).

CQA and flavonoids showed a certain correlation with the stress tolerance of CM plants (Huang, 2010). Importantly, the medicinal quality evaluation indexes of *Chrysanthemum* can be identified from these two categories (Mao, 2020; Chen et al., 2021; Kang et al., 2022). Among the common phytometabolites shared by different medicinal cultivars, compounds with high content and great differences among cultivars, e.g., 4,5-DCQA, flavonoids Acn and Lut, are suitable for screening the index components of quality evaluation, which also have high contribution to CM classification. The content of 4,5- DCQA is high in most cultivars, so it is suitable to be used as a quantitative index component for *Chrysanthemum* quality evaluation. The content of 3-CQA in all samples was high (Mao, 2020), and the difference between cultivars was small. It is suitable to be used as a qualitative index component for *Chrysanthemum* quality evaluation. The low-abundance phytometabolites of *Chrysanthemum* cannot represent its chemical characteristics, e.g., 1-CQA, anthocyanins and carotenoids, which are not suitable for the quality evaluation. The anti-hepatotoxic 1,5-DCQA was at high levels in *C. pacificum* flowers and aerial parts reaching 3,145 and 1,390 μg/g, respectively (Farag et al., 2015), suggesting an alternative natural resource of medicinal compounds.

The correlation analysis between the pharmacologically active CM ingredients showed that there was a very significant positive correlation between chlorogenic acid, isochlorogenic acid B, isochlorogenic acid A and isochlorogenic acid C (Liu, 2020), and chlorogenic acid, isochlorogenic acid B/C and tilianin was significantly positively correlated. There was a very significant positive correlation between isochlorogenic acid C and tilianin, while cynaroside and Lut-7-O-β-D-glucuronic acid had a very significant positive correlation. There was a significant positive association between Dio-7-O-G and linarin, and a very significant positive correlation between the former and tilianin. These interesting results imply some commonly shared biosynthesis pathway of phenolics and flavonoids, as well as coordinated regulation of metabolic networks in *Chrysanthemum*. The phylogenetically related taxa not only have analogous chemical constituents, their biosynthesis pathways and regulatory components, as well as metabolic networks and signal transduction networks, might also be similar.

The anti-inflammatory components of CM could be 3,5-DCQA, 4,5-DCQA, Lut-7-O-β-G, 1,3-DCQA, 3-CQA, and the like (Peng et al., 2019; Mao, 2020). Some of them were induced by jasmonic acid against western flower thrips (Chen et al., 2019). These compounds can be used as indicator components for quality evaluation of CM, which are also useful in studying the chemical relatedness between CM cultivars (Chen et al., 2021), as well as CM and wild relatives. According to the evaluation results of CM grades, 50 kinds of CM on the market, including Gongju, Hangju, Taiju (made from CM bud), etc., were divided into three grades (Mao, 2020), which was positively correlated with the content of index components. The lower the grade, the lower the index component content. This grading provided a reference for the establishment of TCM quality grade evaluation system. Interestingly, in the abundance of CQA, flavonoids and carotenoids, Boju was largely equivalent to Gongju and many cultivars of Hangju (Chen et al., 2021), although the former is phylogenetically distinct from the latter two (Lyu et al., 2008).

Taiju and full bloom CM of the same cultivar were relatively close in the PCA diagram based on index components (Mao, 2020), indicating that there is a great similarity between their chemical components. Among 13 cultivars, Gongju was the most unique. Gongju, Boju and the characteristic cultivar #1 were quite different from others. The clustering analysis based on contents of 13 components largely agreed with PCA results. Most Hangju samples were clustered into one group, and Huaiju, Chuju and some Hangju samples were clustered together, indicating a certain similarity between them. The characteristic cultivars had certain similarities with Boju, suggesting the kinship between them; alternatively, these might be partially explained by similar cultivation conditions, and/or similar terroir factors (Suo et al., 2011; Hao and Liu, 2021).

The cluster analysis based on chlorogenic acid, Lut, 3,5-DCQA, etc., showed that different CM cultivars were basically clustered by cultivar (Xiong, 2014), and the subgroup composed of Fubaiju cultivars “local early-flowering” and “local late-flowering,” derived from Hang Baiju, clustered with Hangju and Gongju. The chemical cluster of Hangju and Gongju was also confirmed by other independent studies (Liu et al., 2019), which is basically consistent with the traditional classification and molecular marker-based classification of CM (Lyu et al., 2008; Mao, 2020).

The content of chlorogenic acid varied greatly among *Chrysanthemum* sensu lato plants, the highest was 0.975 g/100 g (Shao, 2018), and the lowest was only 0.086 g/100 g. After flowering, the content of chlorogenic acid in the plant will increase to a certain extent. The contents of chlorogenic acid, rutinoside, quercetin, Lut, Api in the leaves of *O. taihangensis* were higher than those in flowers, stems and *C. indicum* (Liu, 2013). The contents of chlorogenic acid, rutinoside and quercetin were higher in the stems and leaves, Lut and Api were less abundant in stems, and they were not detected in leaves. In different harvesting periods, the contents of five phenolics in different tissues of *O. taihangensis* were higher than those in corresponding tissues of *C. indicum*. Interestingly, *O. taihangensis* is often locally used as *C. indicum* in folk medicine, and the people in the producing area report that its aroma is strong, and its tea and medicinal value is far superior to that of *C. indicum*.

### Oligosaccharide, polysaccharide

The sugar components of CM contribute to its dual value of food/medicine; the expressions of most glycolysis-, GT-related genes were induced by ultraviolet-B radiation (Yang et al., 2018b). The health-promoting raffinose and 1-kestose were abundant in *C. dichrum* leaves, flower buds, and blooming flowers (Liu et al., 2021a). The immunomodulatory and anti-inflammatory JFP1-1-2, a homogenous polysaccharide of non-medicinal parts of CM, is composed of mannose, glucose and galactose in a molar ratio of 4.53:3.06:1.00 (Tao, 2017); another bioactive polysaccharide JFP1-2-2 is composed of mannose, galactose, glucose, xylose and galacturonic acid in 3.0:2.3:1.2:1.0:1.0.

The content of polysaccharide prepared by membrane separation method of six CM cultivars of Kaifeng, Henan Province varied between 47.8 and 64.3% (Zhao, 2015), while those obtained by alcohol precipitation were 18.9 and 25.3%. The 295 g boiling water-extracted crude polysaccharide CMJA (yield 7.8%) and 96 g alkali-extracted polysaccharide JHB (2.5%) were obtained from 3.8 kg of dried Huaiju (Zheng, 2015). CMJA contained eight homogeneous polysaccharide fractions: CMJA0S1 (110 mg, yield 0.4%), CMJA0S2 (2.2%), CMJA1a S2 (2.3%), CMJA1a S3 (0.4%), CMJA1b S2 (0.5%), CMJA1b S3 (2.0%), CMJA2S2 (2.0%) and CMJA2S3 (2.2%). JHB contained a polysaccharide JHB0S2 (532 mg, yield 1.9%). CMJA0S1 is an arabinogalactan with molecular weight (MW) of 4.7 × 10^4^ Da. The MW of CMJA0S2 is 6.5 × 10^3^ Da; its main chain is a galactomannan glucan composed of 1,4-β-Glcp, 1,4-β-Galp, 1,4-β-Manp, with the branched chains composed of 1,6-β-Galp, T-α-Glcp, T-α-Araf, 1,5-α-Araf, etc. CMJA1a S2, CMJA1a S3, CMJA1b S2, CMJA1b S3 and CMJA2S2 are a series of RG-I type pectin polysaccharides with different MWs, and their galacturonic acid content and branched-chain glycosyl composition varied. 1,2-α-Rhap and 1,4-α-Gal Ap constitute the main chain, and at the O-4 position of rhamnose, there are branched chains composed of arabinose and galactose. CMJA2S3 is a HG-type pectin polysaccharide of 1.1 × 10^4^ Da and composed of galacturonic acid. JHB0S2 is a xyloglucan of 1.6 × 10^4^ Da, with 1,4-β-Glcp constituting the main chain and a branch at the O-6 position, connecting xylose residues.

### Mineral elements

Mineral elements absorbed by *Chrysanthemum* plants play an important role in regulating the processes of primary and secondary metabolism (Liu, 2020). They are the constituent factors of active ingredients in traditional medicine, and have a great impact on the formation and accumulation of bioactive metabolites in TCM plants. CM contained mineral elements such as calcium, magnesium, phosphorus, sulfur, potassium, and indispensable trace elements like copper, iron, zinc, cobalt, manganese, strontium, selenium. The total beneficial mineral elements (K, Ca, Mg, Fe, Na) contained in six CM cultivars of Kaifeng were between 32,008.7 and 40,183.1 mg/kg (Zhao, 2015), while the hazardous As, Pb, Hg and Cd were between 0.01–0.04 and 0.63–1.53 mg/kg. The CM flowers and leaves are rich in N, P, K, Ca, Mg, and Fe (Yan et al., 2021), among which K element had the largest variation, and N, Ca, Fe, Mg, and Zn had a large variation range. The absorption and accumulation of various elements in leaves of different germplasm resources are distinct. There is a strong positive correlation between Ca and Mg/Mn/Cd. The PCA found that CM cultivars were separated based on Mn, Cr, Cu, P, K. From the perspective of mineral elements, Hangju-Fuhuangju, Hangju-Xiaoyangju Late-ripening, Hangju-Sheyangju Late-ripening, Hangju-Dayanghua, Hangju-Subeiju were of good quality. The PCA score map divided the 23 CM samples into four groups, and the cluster analysis heat map divided them into five categories. The samples of the same cultivar clustered together well, indicating that the differences in the mineral element content of CM germplasm resources are closely related to genetic factors.

There was a significant positive correlation between CM isochlorogenic acid A/tilianin and N/P element contents (Liu, 2020; Liu et al., 2021c), isochlorogenic acid B and N element were significantly positively correlated, whereas chlorogenic acid, isochlorogenic acid A, C and cymaroside were significantly negatively correlated with heavy metal Pb. Cynaroside had a very significant negative relationship with Mn; Dio-7-O-G had a very significant positive correlation with Mn and Mg, and had a significant positive correlation with Ca and Pb. There was a significant positive correlation between linarin and Cd/Cr heavy metals. These imply the potential roles of phenolics and flavonoids in *Chrysanthemum* stress/defense response, as well as possible approaches of regulating phytometabolite yields.

### Nutrition constituents

Seventeen amino acids were identified in CM, and contents of Lys, Phe and Leu were relatively high. The contents of seven essential amino acids, i.e., Thr, Val, Met, Ile, Leu, Phe and Lys, in the manufactured goods were 2.3–6 times those in the fresh flowers (Wang et al., 2016). The total amino acids of six CM cultivars of Kaifeng varied between 10.5 and 11.9% (Zhao, 2015), and the essential amino acids were between 2.4 and 3.0%. Asparagine and glutamine were abundant in *C. dichrum* leaves, flower buds, and blooming flowers (Liu et al., 2021a).

Among 28 nucleobases, nucleosides, nucleotides and amino acids of CM of nine geographical origins, eight crucial quality markers, i.e., 2′-deoxyadenosine, guanosine, adenosine 3′,5′-cyclic phosphate (cAMP), guanosine 3′,5′-cyclic monophosphate (cGMP), arginine, proline, glutamate and tryptophan, were identified (Chang et al., 2021), which could be used to discriminate geographical origin of CM.

The crude fat content of six CM cultivars of Kaifeng was between 3.6 and 7.1% (Zhao, 2015). Twenty chemical constituents were detected after methylation of fatty acids, including five saturated fatty acids; unsaturated fatty acids accounted for 69.9% of the total fatty acids. The contents of fatty acids, e.g., capric acid (C10.0), pentadecanoic acid (C15.0), α-linolenic acid (C18.3 N3), eicosanoic acid (C20.0), docosanoic acid (C22.0) and lignoceric acid (C24.0), flavonoids and CQAs were increased in CM by UV-B radiation (Yang et al., 2018b). The contents of moisture, protein, fat, ash and carbohydrate in *C. mongolicum* tea were 8.2, 13.7, 4.1, 6.1 and 67.7%, respectively (Xiao, 2019); all essential amino acids were detected, and the content of amino acids was 11.3 g/100 g. Twenty-two kinds of fatty acids were detected, with abundant linoleic acid (22.8%) and α-linolenic acid (7.4%).

The carotenoids, e.g., zeaxanthin, carotene, cryptoxanthin, lutein, etc., were identified in different CM cultivars (Park et al., 2015; Zhou et al., 2019b; Mao, 2020; Chen et al., 2021). The average total content of carotenoid was highest in the 10% open, followed by 70 and 100% bloom stages at 0.50, 0.43, and 0.36 mg/g FW, respectively. The CM cultivars with higher carotenoid contents displayed yellow or green petal colors (Park et al., 2015). The expression of carotenoid biosynthetic genes in the petals of different CM cultivars at mid-development showed no differences (Kishimoto and Ohmiya, 2006). The antioxidant vitamin C contents of six CM cultivars of Kaifeng varied between 21.7 and 62.7 mg/100 g (Zhao, 2015). CM is beneficial to human health and is expected to be widely developed and applied in plant food, tea making, health care products, and such like. The nutrient composition measurement results were graded (Zhao, 2015), the nutritional index values of each CM cultivar were accumulated to obtain the comprehensive nutritional evaluation index, so as to compare various CM cultivars and select cultivars that are prioritized for human consumption.

When compared with *C. indicum*, quinones, flavonoids (e.g., naringenin 7-O-G, quercetin 4’-O-G), steroids, lipids and carbohydrates were more abundant in *C. nankingense* (Zou et al., 2022), which is usually eaten as a vegetable in Nanjing, China, and its phenylpropanoid, terpene, indole derivatives and alkaloid contents were relatively low. Among identified 477 metabolites, 72 showed significant differences between *C. mongolicum* and *C. rhombifolium* (Duan et al., 2022), mainly flavonoids, organic acids and nucleotides. The metabolomic techniques should be applied in more *Chrysanthemum* species to obtain a holistic and comparative view of their nutritional value.

### Other constituents

**Figure 8:**
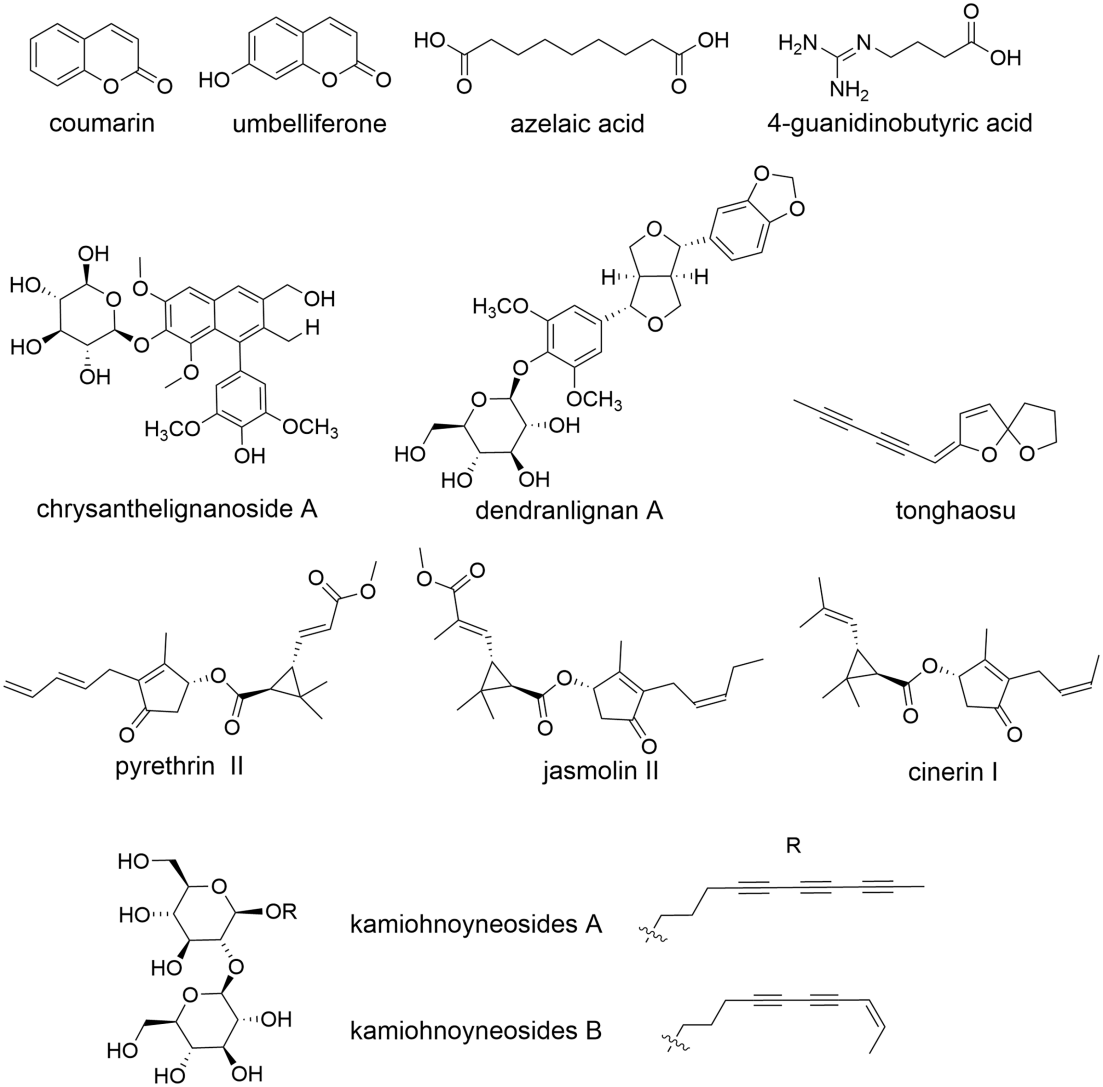
Examples of other phytometabolites of *Chrysanthemum*. Coumarin, umbelliferone, azelaic acid (*C. indicum* Zou et al., 2022), 4-guanidinobutyric acid, chrysanthelignanoside A (neuroprotection, CM), dendranlignan A (anti-inflammatory, CM), tonghaosu, pyrethrin II, jasmolin II, cinerin I, kamiohnoyneosides A and B (anti-diabetic, CM).

Coumarin, umbelliferone, lignan, tonghaosu, pyrethrin, jasmolin, cinerin, etc., were also found in CM (Yang et al., 2019; Zhou et al., 2019b; Mao, 2020; Figure 8). Pyrethrins, a natural insecticide, are biosynthesized by the Anthemideae plants such as *Tanacetum cinerariifolium* (Matsuda, 2012) and CM (Mao, 2020). Their monoterpenoid acid and rethrolone-type oxylipin alcohol moieties are biosynthesized via the 2-C-methyl-D: -erythritol 4-phosphate (MEP) and oxylipin pathways, respectively (Lybrand et al., 2020). The organic acids, e.g., azelaic acid, 4-guanidinobutyric acid, are abundant in *C. indicum* (Zou et al., 2022). The anti-diabetic polyacetylene glycosides from flowers of edible *C. “Kamiohno”* are also reported (Kurimoto et al., 2021).

### Quality control methods and standards

Five kinds of medicinal CM are listed in the Chinese Pharmacopoeia (2020), including Boju, Hangju, Chuju, Huaiju, Gongju. The diversity of species, origins, cultivation, processing and harvesting methods directly affects the medicinal components and effects. However, the traditional HPLC method of measuring the contents of chlorogenic acid, Lut-7-O-G, 3,5-DCQA is not enough to evaluate the quality of medicinal CM comprehensively. The concept of quality marker (Q-marker) provides a new idea for quality control of TCM. Q-marker is based on the perspective of biological activity, and various methods are utilized to find the chemical components that best reflect biological effects of TCM (Liu et al., 2018). Multiple methods, especially multi-ingredients quantification, chromatographic fingerprint and/or their combination (Nie et al., 2019; Peng et al., 2019), have been applied extensively in the quality control of CM. In recent years, quality verification methods based on the activity-integrated fingerprints have received wide attention. For instance, an antioxidant activity based method combining the online HPLC-DPPH, ESI-MS, and NIR spectra analysis identified 16 antioxidants of five CM cultivars (Zhang et al., 2022). Phenolic acids play a more important role in antioxidant activity, and chlorogenic acid, Lut-7-O-G, 3,5-DCQA, 4,5-DCQA were observed as the main contributors to the overall antioxidant capacity. The chromatographic fingerprint and *in vitro* antioxidant activity assay were combined to show that chlorogenic acid, 3,5-DCQA, 1, 4,5-O-DCQA and kaempferol-3-O-rutinoside could be Q-markers of Hang-baiju, Gongju, Huaiju, Taiju and Boju (Lu et al., 2022b). In the future, the strategy based on activity-integrated fingerprints has great potential in quality control of traditional medicine, food and other fields, as it can fully reflect the pharmacological information of active ingredients.

## Pharmacological properties

**Figure 9:**
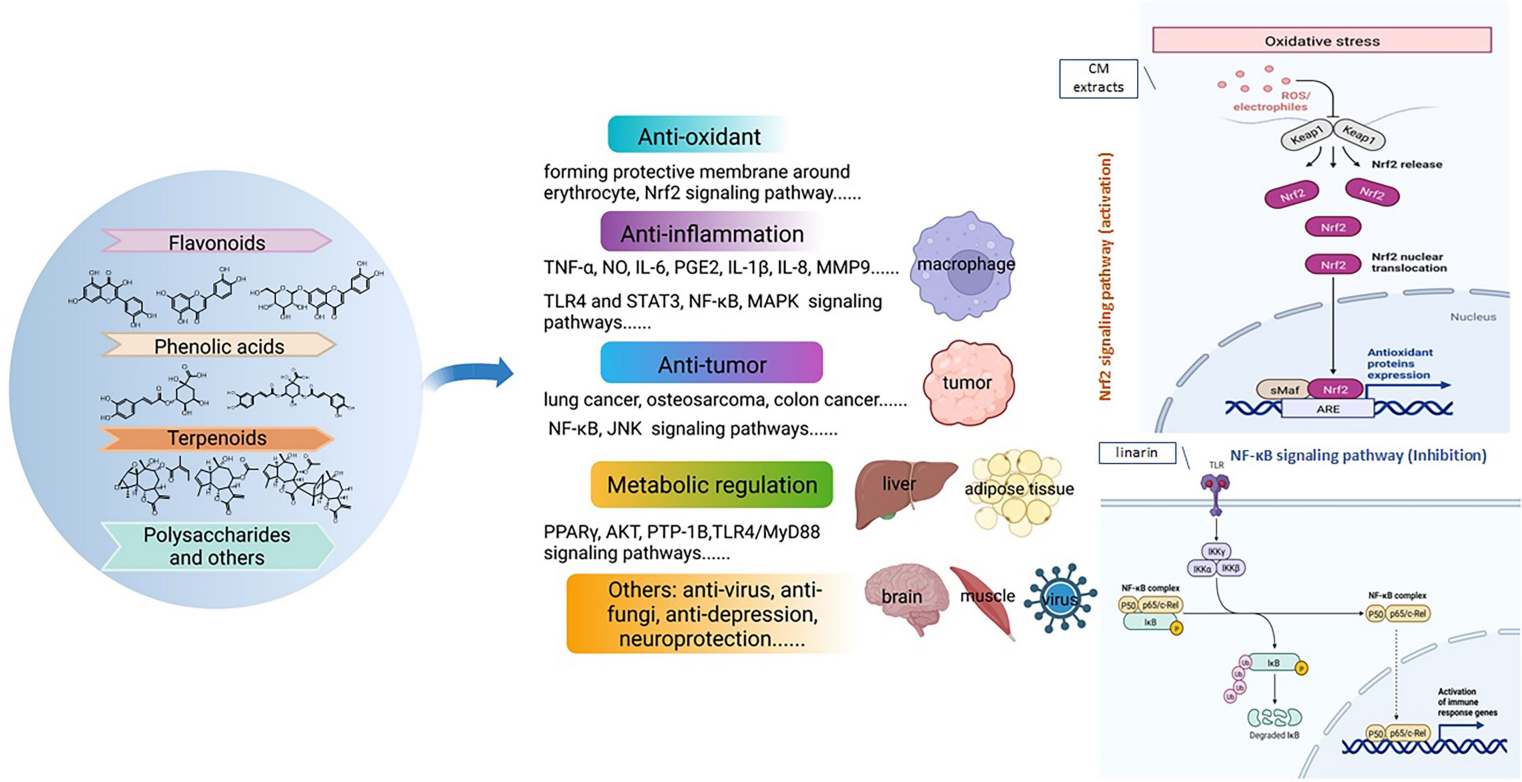
Illustration of pharmacological mechanisms exerted by *Chrysanthemum* extracts and compounds. Various bioactive compounds produced in *Chrysanthemum* plants are shown in left panel; their diverse pharmacological activities and possible mechanisms of action are exemplified in the central part; two representative signaling pathways regulated by *Chrysanthemum* components are shown on the right part. ARE: Antioxidant response element; NF-κB: Nuclear factor kappa-B; Nrf2: NF-E2-related factor 2; TLR4: Toll-like receptor 4.

The plants of *Chrysanthemum* and related taxonomic groups are traditionally used as ethnomedicine (Figure 1; Jia and Zhang, 2016). *Chrysanthemum* plants have a wide range of pharmacological activities (Figure 9; Supplementary Table S1). In recent years, the main studied taxa include CM (Khan et al., 2020), *C. indicum* (Tian et al., 2020), *C. boreale* (Kim et al., 2022) and *C. zawadskii* (Kim et al., 2019), among others. The most reported activity of *Chrysanthemum* is its anti-inflammatory and immunomodulatory effects (Xue et al., 2021; Kim et al., 2022), which are consistent with traditional efficacy; it also shows great potential for improving chronic metabolic diseases, neurodegenerative diseases, etc. As the main active ingredients, flavonoids, phenolic acids and terpenoids may be the principal material basis for these effects. For example, total flavonoids of CM cultivar Bianliang ziyu prevented hepatotoxicity by inhibiting oxidative stress and apoptosis via the activation of Nrf2 signaling (Tian et al., 2019); caffeoylquinic acids, chlorogenic acid, gallocatechin, Lut-7-OG, Acn-7-O-rutinoside, and anthocyanins of *Chrysanthemum* showed strong antioxidant activities (Supplementary Table S1.). Acn-7-O-rutinoside, Lut-7-OG, and chlorogenic acid also showed the anti-inflammatory activity (Zhang et al., 2019). Flavonoids (including linarin, Dio-7-G, tilianin, etc.) and phenolic acids (including isochlorogenic acid C, isochlorogenic acid A, 1,3-DCQA, etc.) of *Chrysanthemum* improved the inflammatory bowel disease of zebrafish by regulating the expressions of IL-1β, IL-8 and MMP9 (Li et al., 2022). Eriodictyol-7-O-β-d-glucuronide and 5,7-dihydroxy-4-chromene of *C. zawadskii* var. *latilobum* had antiallergic effects in FcεRI-mediated human basophilic KU812F cells (Lee and Shim, 2020). The activities of *Chrysanthemum* flavonoids in metabolic regulation are also salient. For instance, Lut and luteoloside improved blood lipids and hepatic steatosis in hyperlipidemia rats by regulating antioxidant levels and lipid metabolism (Sun et al., 2021); naringenin and naringenin-7-O-G inhibited the intracellular lipid accumulation by the activation of PPARγ and phosphorylation of the PI3K/Akt pathway (Nishina et al., 2019); Lut, Acn, and buddleoside controlled the postprandial glucose concentration by inhibiting the activity of α-amylase (Li et al., 2019). Other activities of *Chrysanthemum* flavonoids are also intriguing. Linarin and scutellarein displayed anticancer activity (Jung et al., 2019; Li et al., 2020); buddleoside reduced blood pressure in spontaneously hypertensive rats by inhibiting the vascular TLR4/MyD88 pathway and improving vascular endothelial function (Wang et al., 2021c); Acn-7-O-β-D-rutinoside prevented dexamethasone-evoked muscle atrophy via the Akt/mTOR pathway and decreasing the mitochondrial respiration (Lee et al., 2021).

When compared with flavonoids and phenolics, the antioxidant activity and metabolic regulation of *Chrysanthemum* terpenoids are less reported (Supplementary Table S1.), but their anti-inflammatory, anticancer and other properties are remarkable. For example, chrysanthemulide A (sesquiterpenoid) of *C. indicum* induced apoptosis of osteosarcoma by upregulating death receptor 5 via JNK-mediated autophagosome accumulation (Zhuo et al., 2019); it also showed the anti-inflammatory activity via suppressing the LPS-induced NF-κB pathway and down-regulating MAPK activation (Xue et al., 2018); cumambrin A (sesquiterpenoid) suppressed the osteoclast formation, bone resorption, and RANKL-induced signaling pathways in the treatment of osteoporosis (Zhou et al., 2019a). The recently discovered sesquiterpenes are subject to the in vitro activity screening (Jiang et al., 2021a), whose efficacies and corresponding mechanisms of action warrant further explorations.

The activities of *Chrysanthemum* polysaccharides have also attracted attention (Wang et al., 2021b), e.g., antioxidant, anti-inflammatory and anti-viral (Supplementary Table S1), and other compounds and extracts exhibited diversified bioactivities. The antimicrobial, anti-inflammatory, anti-hypertension and antioxidant effects (Supplementary Table S1) of CM have been utilized by ancient doctors and herbalists to help maintain body balance and relieve much of the sufferings. The other tantalizing effects of CM components, e.g., anti-aging and anti-diabetic, are being revealed by contemporary polypharmacology investigations, which facilitate drug repurposing and benefit more people. The phylogenetically related *Chrysanthemum* species are more likely to possess similar chemical profiles, despite the impact of environmental factors. The most typical examples are CM and wild relatives. The medicinal compounds and therapeutic efficacy of CM are closer to *C. indicum* than to *C. zawadskii*, and are more dissimilar to those of *C. boreale*. Significant or subtle differences in chemical composition between closely related species could be useful in tailoring personalized therapeutic protocol for each patient.

## Conclusions and prospects

This article summarized the phylogeny, biodiversity, phytochemistry, and chemodiversity of *Chrysanthemum*, especially the cultivated hybrid CM. As a unique Chinese culture, the chrysanthemum culture has witnessed the development of history and had a profound influence on human health. The ethnomedicinal experiences, phytochemistry and bioactivity studies are the prerequisite of product research and development; to date more than 120 CM compounds have been isolated and identified, and these monomeric compounds and crude extracts are screened for pharmacological activities *in vivo* and in vitro. The experimental studies validated the traditional medicinal uses of CM, but the responsible chemicals have not been thoroughly determined. Thus, there is a need of bioassay-guided identification of the bioactive components. What’s more, the relationship between traditional uses and recent pharmacological findings is not always clear, and it is imperative to investigate the biochemical and physiological mechanisms of components of CM and evolutionarily related taxa, especially their cardiovascular protection, anti-tumor and antioxidant activities. Efforts should also be made to determine the modes of action, bioavailability, pharmacokinetics and physiological pathways of specific functional compounds in *Chrysanthemum* and related taxonomic groups. Moreover, clinical studies, e.g., randomized controlled trial, should be encouraged to identify any side effects and possible interactions between *Chrysanthemum* herbal medicine and other natural medicines/synthetic drugs. Further safety verification and clinical trials should be carried out to expand the application scope of CM and better integrate it into medicinal practice. With multiple chemical ingredients, CM may exert its beneficial effects by gently interacting with different cell signaling pathways and networks, which achieve the same therapeutic efficacy as that of mono-ingredient agents, whereas CM doses are much lower than those of single compounds. In order to better mine the medicinal potential and edible value of different *Chrysanthemum* cultivars and Daodi medicinal materials, the intricate link between phylogenetic relationship, chemical profile, ethnomedicinal knowledge and pharmacological activities should be scrutinized within the pharmacophylogenetic framework (Hao and Xiao, 2017, 2020).

The emerging pharmacophylogenomics (Hao and Xiao, 2017, 2020) calls for much more genomic data of *Chrysanthemum* and related taxonomic groups. The rapid development of reduced-representation genome sequencing enables the cost-effective sequencing of Asteraceae plant (Mérot, 2020). It is of low cost and strong sequencing performance. In the future, it can be used in *Chrysanthemum* phylogeny, plant identification and genome assisted breeding. The cp genome with maternal genetic characteristics has the advantages of multi-copy and conservative structure. The combination of cp genome and high-throughput sequencing technology has become an effective means of plant genetic resources research, which facilitates the study of *Chrysanthemum* classification and genetic resources (Tyagi et al., 2020; Masuda et al., 2022). The accumulation of whole genome sequencing data of *Chrysanthemum* (Song et al., 2018; Nakano et al., 2021; van Lieshout et al., 2022) and relevant taxonomic groups will enable the comprehensive phylogenomic analyses to reveal the hybridization/polyploidization events leading to the speciation and formation of CM. The assembly and annotation of more high-quality *Chrysanthemum* genomes will help to elucidate the genus evolution and its contributions to gene abundance/function.

The genome editing enables the induction of mutations in a targeted genomic region and has recently played a substantial role in Asteraceae functional genomics and biotechnology (Bernard et al., 2019; Park et al., 2019). As precise mutations can be generated in the targeted sequence, it is considered to be more effective than conventional mutation breeding. This breakthrough technology has been adopted for various Asteraceae crops such as chicory and lettuce (Bernard et al., 2019; Park et al., 2019), and could be used in *Chrysanthemum* for desired phenotypes. Unlike transgenic technology, genome editing does not require the transgene to be integrated with the genome, and the integrated transgene could be segregated in the progeny.

At present, there are very few reports on the use of metabolomics in *Chrysanthemum* taxa. The metabolomics technology can be used to comprehensively characterize the chemical components of *Chrysanthemum* species and varieties, obtain quantitative information of each phytometabolite, and quickly screen the characteristic chemical, so as to provide effective data for the quality evaluation and control of *Chrysanthemum* samples, and contribute new ideas and references for the elucidation of medicinal material basis of *Chrysanthemum*. The evaluation and control provide effective data, inspire new ideas and contribute references for the elucidation of the medicinal material basis of chrysanthemum. The metabolic research is conducive to the realization of the integrity and traceability of chrysanthemum quality control. The metabolic makeup of fresh flowers and processed products may vary greatly; how to achieve the transferability of chrysanthemum quality in multiple links such as harvesting, crude processing, fine processing, and production of patent medicines still needs to be studied from multiple hierarchies. However, the relative quantification using the peak area as an index cannot reflect the exact content of compounds in chrysanthemum, therefore it is necessary to establish the quantitative analysis method for the differential components of different species/varieties of *Chrysanthemum*. The compounds detected by UPLC-Q-TOF-MS in plant metabolomics are more comprehensive (Hao et al., 2021). To find the quality control indicators of chrysanthemum from numerous compounds, it is obligatory to combine content determination and pharmacokinetics/pharmacodynamics for comprehensive evaluation, which warrant deeper studies in the pharmacologically active constituents of chrysanthemum.

The current highly intensive industrial systems of agricultural and horticultural production are counter-sustainable, as the energy consumption is particularly intensive for cultivation, and the excessive use of nitrogen fertilizer leads to the worrying emission of greenhouse gas, N_2_O (Hao et al., 2021; Wang et al., 2022). The genetic modification (GM) is transforming the prospects of sustainable crop protection, the specialized metabolite (e.g., pyrethrins) biosynthesis genes can be integrated to the target *Chrysanthemum* plants to enhance the stress response and disease resistance, so as to realize the full potential of GM and offer greener crop protection.

This article applies the systematic and holistic characteristics of plant pharmacophylogeny to the regular arrangement of biodiversity and chemodiversity explorations on *Chrysanthemum*. On the basis of scientific and technological innovation, and with the integration of holistic view of traditional medicine, we have deepened our understanding of the following points: The phylogenetically close taxa are more likely to have similar metabolic profile, and the similar metabolic makeup could result in analogous pharmacokinetic behavior and clinical efficacy (Hao and Xiao, 2020). The studies of pharmacophylogeny are conducive to the sustainable conservation and rational utilization of *Chrysanthemum* resources, as well as the inheritance and innovation of traditional medicine. The medicinal properties of plants can be predicted by virtue of phylogenetic methods (Hao et al., 2022a,b), which has been utilized to explore the regularity of bioactivities of Asteraceae and Ranunculales plants against diseases of multiple human organs. The mapping of different types of *Chrysanthemum* (and related taxonomic groups) compounds onto the species phylogenetic tree could also be beneficial to mining novel sources of economically important chemicals. Disentangling recent speciation events and distribution of compounds/therapeutic effects usually requires a reliable phylogenetic framework, therefore research in phylogeny/evolution of *Chrysanthemum* and related taxonomic groups cannot be overemphasized. Besides the intensive studies of CM, the evaluation of other *Chrysanthemum* species should also keep pace with the times and develop scientifically. The ability to correlate the chrysanthemum metabolome with the medicinal efficacy in the context of pharmacophylogeny will help to develop chrysanthemum cultivars with better curative effect and more commercial value in the near future.

## Author contributions

D-CH and PX: conceptualization and writing—review and editing. D-CH, YS, and YZ: methodology and analysis. PX and LX: resources and supervision. D-CH, YS, and PW: data curation and visualization. D-CH and YS: writing—original draft preparation. All authors contributed to the article and approved the submitted version.

## Funding

This work is supported by the Scientific Research Funds Project of Liaoning Education Department (JDL2019012), China Scholarship Council (202108210156), CAMS Innovation Fund for Medical Sciences (CIFMS 2021-I2M-1-032), and Hainan Academician Innovation Platform Scientific Research Project and National Science & Technology Fundamental Resources Investigation Program of China (2018FY100700).

## Conflict of interest

The authors declare that the research was conducted in the absence of any commercial or financial relationships that could be construed as a potential conflict of interest.

## Publisher’s note

All claims expressed in this article are solely those of the authors and do not necessarily represent those of their affiliated organizations, or those of the publisher, the editors and the reviewers. Any product that may be evaluated in this article, or claim that may be made by its manufacturer, is not guaranteed or endorsed by the publisher.

## Abbreviations

Acn: Acacetin
AFLP: Amplified fragment length polymorphism
AG: Acetylglucoside
ANS: Anthocyanidin synthase
Api: Apigenin
CDDP: Conserved DNA-derived polymorphism
CHI: Chalcone isomerase
CHS: Chalcone synthase
CIF: Chrysanthemi Indici Flos
CM: Chrysanthemum× morifolium
cp: chloroplast
CYP: Cytochrome p450
DCQA: Dicaffeoylquinic acid
DFR: Dihydroflavanol 4-reductase
Dio: Diosmetin
EST: Expressed sequence tag
F3′H: Flavonoid 3′-hydroxylase
F3′5′H: Flavonoid 3’5’-hydroxylase
FISH: Fluorescence *in situ* hybridization
GT: Glycosyltransferase
IGS: Intergenic spacer
ISSR: Inter-simple sequence repeat
Lut: Luteolin
MG: Malonylglucoside
MW: Molecular weight
nr ITS: Nuclear ribosomal internal transcribed spacer
PCA: Principal component analysis
QTP: Qinghai-Tibetan Plateau
RAPD: Random amplified polymorphic DNA
RFLP: Restriction fragment length polymorphism
SCAR: Sequence-characterized amplified region
SNP: Single nucleotide polymorphism
SSR: Simple sequence repeat
TCM: Traditional Chinese medicine
TPS: Terpene synthase
TS: Terpenoid synthase
UGT: UDP-glucuronosyl and -glucosyltransferase
UPGMA: Unweighted pair group method with arithmetic means
WGD: Whole genome duplication

## References

[ref1] Beninger C. W. Abou-Zaid M. M. Kistner A. L. Hallett R. H. Iqbal M. J. Grodzinski B.. (2004). A flavanone and two phenolic acids from *Chrysanthemum morifolium* with phytotoxic and insect growth regulating activity. J. Chem. Ecol. 30, 589–606. doi: 10.1023/B:JOEC.0000018631.67394.e5, PMID: 15139310

[ref2] Bernard G. Gagneul D. Alves Dos Santos H. Etienne A. Hilbert J. L. Rambaud C. (2019). Efficient genome editing using CRISPR/Cas9 technology in chicory. Int. J. Mol. Sci. 20:1155. doi: 10.3390/ijms20051155, PMID: 30845784 PMC6429391

[ref3] Cai Y. Gao Y. Zhang Z. Liu H. Wang Y. Ma Y.. (2022). Development and application of a cultivar-specific sequence-characterized amplified region (SCAR) marker for the detection of *Chrysanthemum morifolium* Ramat. 'Daboju'. Plants. 11:604. doi: 10.3390/plants11050604, PMID: 35270074 PMC8912837

[ref4] Chang X. W. Wei D. D. Chen D. J. Yan H. Sun X. D. Zhu W. B.. (2019). Historical origin and development of medicinal and tea *Chrysanthemum morifolium* resource. Modern Chin. Med. 21, 116–123. doi: 10.13313/j.issn.1673-4890.20181224005

[ref5] Chang X. Zhang Z. Yan H. Su S. Wei D. Guo S.. (2021). Discovery of quality markers of nucleobases, nucleosides, nucleotides and amino acids for Chrysanthemi Flos from different geographical origins using UPLC-MS/MS combined with multivariate statistical analysis. Front. Chem. 9:689254. doi: 10.3389/fchem.2021.689254, PMID: 34422760 PMC8375154

[ref600] Chen J. Y. (2005). Contributions of Chinese Chrysanthemum to the world in the past and future. J. Chin. Landscape Architect. 9, 73–75.

[ref6] Chen G. Kim H. K. Klinkhamer P. G. Escobar-Bravo R. (2019). Site-dependent induction of jasmonic acid-associated chemical defenses against western flower thrips in Chrysanthemum. Planta 251:8. doi: 10.1007/s00425-019-03292-2, PMID: 31776674

[ref7] Chen T. Li L. P. Lu X. Y. Jiang H. D. Zeng S. (2007). Absorption and excretion of luteolin and apigenin in rats after oral administration of *Chrysanthemum morifolium* extract. J. Agri. Food Chem. 55, 273–277. doi: 10.1021/jf062088r, PMID: 17227053

[ref8] Chen S. Liu J. Dong G. Zhang X. Liu Y. Sun W.. (2021). Flavonoids and caffeoylquinic acids in *Chrysanthemum morifolium* Ramat flowers: A potentially rich source of bioactive compounds. Food Chem. 344:128733. doi: 10.1016/j.foodchem.2020.128733, PMID: 33280963

[ref9] Chen X. J. Sun M. Liang J. G. Xue H. Zhang Q. X. (2013). Genetic diversity of species of Chrysanthemum and related genera and groundcover cultivars assessed by amplified fragment length polymorphic markers. HortScience 48, 539–546. doi: 10.21273/HORTSCI.48.5.539

[ref601] Chinese Pharmacopoeia Commission. (2015). Pharmacopoeia of the People’s Republic of China. Beijing: China Medical Science Press, 1, 310–311.

[ref10] Chinese Pharmacopoeia Commission. (2020). Pharmacopoeia of the People’s Republic of China. Βeijing: China Medical Science Press, 1, 323–324.

[ref11] Chong X. Zhang F. Wu Y. Yang X. Zhao N. Wang H.. (2016). A SNP-enabled assessment of genetic diversity, evolutionary relationships and the identification of candidate genes in Chrysanthemum. Genome Biol. Evol. 8, 3661–3671. doi: 10.1093/gbe/evw270, PMID: 28082602 PMC5521737

[ref12] Clifford M. N. Wu W. Kirkpatrick J. Kuhnert N. (2007). Profiling the chlorogenic acids and other caffeic acid derivatives of herbal Chrysanthemum by LC-MSn. J. Agri. Food Chem. 55, 929–936. doi: 10.1021/jf062314x, PMID: 17263495

[ref13] Criado Ruiz D. Villa Machío I. Herrero Nieto A. Nieto Feliner G. (2021). Hybridization and cryptic speciation in the Iberian endemic plant genus Phalacrocarpum (Asteraceae-Anthemideae). Mol. Phylogenet. Evol. 156:107024. doi: 10.1016/j.ympev.2020.107024, PMID: 33271372

[ref14] Cui N. X. (2004). Meiosis behavior and genetic relationship of several Chrysanthemum plants and their hybrids. MSc thesis. [Nanjing(Jiangsu)]: Nanjing Agricultural University, 1–61.

[ref15] Dai Z. Li S. X. Chen S. Q. Zhu Y. (2017). Quality evaluation of Huaiju germplasm resources. Chin. J. Expt. Trad. Med. Formulae. 23, 48–54. doi: 10.13422/j.cnki.syfjx.2017060048

[ref16] Ding L. Chen F. D. Teng N. J. Fang W. M. Zhao J. Y. (2008). Isozyme analysis of wild and different purpose Chrysanthemum. J. Nanjing Agri. Univ. 2008, 37–42.

[ref17] Duan X. X. Zhang W. J. Li J. J. Xu H. Hu J. Zhao L.. (2022). Comparative metabolomics analysis revealed biomarkers and distinct flavonoid biosynthesis regulation in Chrysanthemum mongolicum and C. rhombifolium. Phytochem. Anal. 33, 373–385. doi: 10.1002/pca.3095, PMID: 34750870

[ref19] Fan M. Gao Y. K. Gao Y. H. Wu Z. Liu H. Zhang Q. X. (2019). Characterization and development of EST-SSR markers from transcriptome sequences of chrysanthemum (Chrysanthemum× morifolium Ramat.). Hort Science 54, 772–778. doi: 10.21273/HORTSCI13694-18

[ref20] Farag N. F. Farag M. A. Abdelrahman E. H. Azzam S. M. El-Kashoury E. S. A. (2015). Metabolites profiling of Chrysanthemum pacificum Nakai parts using UPLC-PDA-MS coupled to chemometrics. Nat. Prod. Res. 29, 1342–1349. doi: 10.1080/14786419.2015.1025396, PMID: 25810048

[ref21] Feng S. G. He R. F. Lu J. Jiang M. Shen X. Jiang Y.. (2016). Development of SSR markers and assessment of genetic diversity in medicinal *Chrysanthemum morifolium* cultivars. Front. Genet. 7:113. doi: 10.3389/fgene.2016.00113, PMID: 27379163 PMC4908101

[ref602] Flora Reipublicae Popularis Sinicae. (1983). Beijing: Science Press, 76, 35–39.

[ref22] Hao D. C. Chen S. L. Osbourn A. Kontogianni V. G. Liu L. W. Jordán M. J. (2015). Temporal transcriptome changes induced by methyl jasmonate in *Salvia sclarea*. Gene 558, 41–53. doi: 10.1016/j.gene.2014.12.043, PMID: 25536164

[ref23] Hao D. C. Li P. Xiao P. G. He C. N. (2021). Dissection of full-length transcriptome and metabolome of Dichocarpum (Ranunculaceae): implications in evolution of specialized metabolism of Ranunculales medicinal plants. PeerJ. 9:e12428. doi: 10.7717/peerj.12428, PMID: 34760397 PMC8574218

[ref24] Hao D. C. Liu C. X. (2021). Back to beginning: Searching for Rosetta Stone of enhancing herbal medicine quality. Chin. Herb. Med. 13, 299–300. doi: 10.1016/j.chmed.2022.03.005 PMC9476797 36118925

[ref25] Hao D. C. Lyu H. Y. Wang F. Xiao P. G. (2022a). Evaluating potentials of species rich taxonomic groups in cosmetics and dermatology: Clustering and dispersion of skin efficacy of Asteraceae and Ranunculales plants on the species phylogenetic tree. Curr. Pharm. Biotech. 23:926. doi: 10.2174/1389201023666220324123926, PMID: 35331107

[ref27] Hao D. C. Xiao P. G. (2017). An Introduction of Plant Pharmacophylogeny. Beijing: Chemical Industry Press.

[ref28] Hao D. C. Xiao P. G. (2020). Pharmaceutical resource discovery from traditional medicinal plants: Pharmacophylogeny and pharmacophylogenomics. Chin. Herb. Med. 12, 104–117. doi: 10.1016/j.chmed.2020.03.002, PMID: 36119793 PMC9476761

[ref29] Hao D. C. Zhang Y. L. He C. N. Xiao P. G. (2022b). Distribution of therapeutic efficacy of Ranunculales plants used by ethnic minorities on the phylogenetic tree of Chinese species. Evid. Based Complement. Altern. Med. 2022, 9027727. doi: 10.1155/2022/9027727 PMC8769838 35069772

[ref30] Hong G. (2014). Preliminary study on intergeneric hybridization of Chrysanthemum and its related genera (VIII). MSc thesis. [Beijing]: Beijing Forestry University, 1–56.

[ref31] Hong G. Wu X. B. Liu Y. C. Xie F. Liu Z. H. Liu W. C.. (2015). Intergeneric hybridization between Hippolytia kaschgarica (Krascheninnikov) Poljakov and *Nipponanthemum nipponicum* (Franch. ex Maxim.) Kitam. Genet. Resour. Crop. Evol. 62, 255–263. doi: 10.1007/s10722-014-0150-1

[ref32] Huang Y. (2010). Preliminary study on cross breeding of medicinal Chrysanthemum. MSc thesis. [Nanjing(Jiangsu)]: Nanjing Agricultural University, 1–87.

[ref33] Huang Z. Liu Z. Y. Wang S. L. Xue Y. Q. Xue J. Q. Zhang X. X. (2020). Resources investigation of Chinese medicinal *Chrysanthemum morifolium* varieties and its industry status analysis. J. Chin. Medi. Materia. 43, 1325–1329. doi: 10.13863/j.issn1001-4454.2020.06.007

[ref34] Jia M.R. Zhang Y. (2016). Dictionary of Chinese Ethnic Medicine. Beijing: China Medical Science Press.

[ref35] Jiang S. Wang M. Jiang Z. Zafar S. Xie Q. Yang Y.. (2021a). Chemistry and pharmacological activity of sesquiterpenoids from the Chrysanthemum genus. Molecules 26:3038. doi: 10.3390/molecules26103038 34069700 PMC8161347

[ref36] Jiang H. Xia Q. Xu W. Zheng M. (2004). *Chrysanthemum morifolium* attenuated the reduction of contraction of isolated rat heart and cardiomyocytes induced by ischemia/reperfusion. Pharmazie 59, 565–567. 15296097

[ref37] Jiang B. P. Xu L. J. Wang Q. L. Xiao P. G. (2013). *Chrysanthemum morifolium*--- Ethnopharmacological use, phytochemistry and pharmacological activity. Modern Chin. Med. 15, 523–530. doi: 10.13313/j.issn.1673-4890.2013.06.016

[ref38] Jiang Y. F. Zhang W. B. Chen X. L. Wang W. Köllner T. G. Chen S.. (2021b). Diversity and biosynthesis of volatile terpenoid secondary metabolites in the Chrysanthemum genus. Criti. Rev. Plant Sci. 40, 422–445. doi: 10.1080/07352689.2021.1969504

[ref39] Jo Y. D. Ryu J. Kim Y. S. Kang K. Y. Hong M. J. Choi H. I.. (2020). Dramatic increase in content of diverse flavonoids accompanied with down-regulation of F-box genes in a Chrysanthemum (Chrysanthemum × morifolium (Ramat.) Hemsl.) mutant cultivar producing dark-purple ray florets. Genes. 11:865. doi: 10.3390/genes11080865, PMID: 32751443 PMC7464468

[ref40] Jung C. H. Han A. R. Chung H. J. Ha I. H. Um H. D. (2019). Linarin inhibits radiation-induced cancer invasion by downregulating MMP-9 expression via the suppression of NF-κB activation in human non-small-cell lung cancer A549. Nat. Prod. Res. 33, 3582–3586. doi: 10.1080/14786419.2018.1484460 29897257

[ref41] Kang S. H. Nie J. Chen K. L. Deng H. Y. (2022). Comparison of morphological characteristics and quality evaluation of *Chrysanthemum morifolium*. J. Chin. Medi. Mate. 2022, 49–57. doi: 10.13863/j.issn1001-4454.2022.01.009

[ref42] Khan I. A. Xu W. Wang D. Yun A. Khan A. Zongshuai Z.. (2020). Antioxidant potential of *chrysanthemum morifolium* flower extract on lipid and protein oxidation in goat meat patties during refrigerated storage. J. Food Sci. 85, 618–627. doi: 10.1111/1750-3841.15036, PMID: 32052442

[ref43] Kim K. Y. Oh T. W. Yang H. J. Kim Y. W. Ma J. Y. Park K. I. (2019). Ethanol extract of Chrysanthemum zawadskii Herbich induces autophagy and apoptosis in mouse colon cancer cells through the regulation of reactive oxygen species. BMC Complement. Altern. Med. 19:274. doi: 10.1186/s12906-019-2688-0 31638961 PMC6805551

[ref44] Kim D. Y. Won K. J. Hwang D. I. Kim N. Y. Kim B. Lee H. M. (2022). 1-Iodohexadecane alleviates 2,4-dinitrochlorobenzene-induced atopic dermatitis in mice: Possible involvements of the skin barrier and mast cell SNARE proteins. Molecules 27:1560. doi: 10.3390/molecules27051560, PMID: 35268661 PMC8911872

[ref45] Kishimoto S. Ohmiya A. (2006). Regulation of carotenoid biosynthesis in petals and leaves of chrysanthemum (*Chrysanthemum morifolium*). Physiologia Plant. 128, 436–447. doi: 10.1111/j.1399-3054.2006.00761.x, PMID: 23643306

[ref46] Kurimoto S. I. Fujita H. Kawaguchi S. Sasaki Y. F. Nakamura T. Kubota T. (2021). Kamiohnoyneosides A and B, two new polyacetylene glycosides from flowers of edible Chrysanthemum "Kamiohno". J. Nat. Med. 75, 167–172. doi: 10.1007/s11418-020-01443-4, PMID: 32803654

[ref47] Lai J. P. Lim Y. H. Su J. Shen H. M. Ong C. N. (2007). Identification and characterization of major flavonoids and caffeoylquinic acids in three Compositae plants by LC/DAD-APCI/MS. J. Chromatography B. 848, 215–225. doi: 10.1016/j.jchromb.2006.10.028, PMID: 17084113

[ref48] Lee H. Kim Y. I. Nirmala F. S. Jeong H. Y. Seo H. D. Ha T. Y.. (2021). Chrysanthemum zawadskil Herbich attenuates dexamethasone-induced muscle atrophy through the regulation of proteostasis and mitochondrial function. Biomed. Pharmacother. 136:111226. doi: 10.1016/j.biopha.2021.111226, PMID: 33485066

[ref49] Lee M. Shim S. Y. (2020). Inhibitory effects of eriodictyol-7-O-β-d-glucuronide and 5,7-dihydroxy-4-chromene isolated from Chrysanthemum zawadskii var. latilobum in FcεRI-mediated human basophilic KU812F cell activation. Molecules 25, 994. doi: 10.3390/molecules25040994, PMID: 32102220 PMC7070965

[ref50] Li H. (1993). Chinese Chrysanthemum. Nanjing: Jiangsu Scientific and Technological Publishing Co.

[ref51] Li T. (2014). Construction of CDDP fingerprints and genetic diversity analysis of Chrysanthemum. MSc thesis. [Taian(Shandong)]: Shandong Agricultural University, 1–64.

[ref52] Li P. Huang Z. She Y. Qin S. Gao W. Cao Y.. (2019). An assessment of the interaction for three *Chrysanthemum indicum* flavonoids and α-amylase by surface plasmon resonance. Food Sci. Nutri. 8, 620–628. doi: 10.1002/fsn3.1349 PMC6977516 31993185

[ref53] Li Y. Liu X. J. Su S. L. Yan H. Guo S. Qian D. W.. (2022). Evaluation of anti-inflammatory and antioxidant effects of Chrysanthemum stem and leaf extract on zebrafish inflammatory bowel disease model. Molecules 27, 2114. doi: 10.3390/molecules27072114, PMID: 35408512 PMC9000279

[ref54] Li B. Ma C. Zhao X. Hu Z. Du T. Xu X.. (2018). YaTCM: Yet another traditional Chinese medicine database for drug discovery. Comput. Struct. Biotechnol. J. 16, 600–610. doi: 10.1016/j.csbj.2018.11.002, PMID: 30546860 PMC6280608

[ref55] Li J. Wan Q. Abbott R. J. Rao G. Y. (2013). Geographical distribution of cytotypes in the *Chrysanthemum indicum* complex as evidenced by ploidy level and genome-size variation. J. Syst. Evol. 51, 196–204. doi: 10.1111/j.1759-6831.2012.00241.x

[ref56] Li J. Wan Q. Guo Y. P. Abbott R. J. Rao G. Y. (2014). Should I stay or should I go: biogeographic and evolutionary history of a polyploid complex (*Chrysanthemum indicum* complex) in response to Pleistocene climate change in China. New Phytol. 201, 1031–1044. doi: 10.1111/nph.12585, PMID: 24400906

[ref57] Li Y. Wang J. Zhong S. Li J. Du W. (2020). Scutellarein inhibits the development of colon cancer via CDC4-mediated RAGE ubiquitination. Int. J. Mol. Med. 45, 1059–1072. doi: 10.3892/ijmm.2020.4496, PMID: 32124957 PMC7053863

[ref58] Li D. L. Zhu H. W. Ren Q. J. Xu Z. L. (2010). Comparative analysis on the configuration of vegetative organs of medicinal Chrysanthemum from different original locations and species. Zhong Yao Cai 33, 1845–1849. doi: 10.13863/j.issn1001-4454.2010.12.005 21548357

[ref59] Liu R. (2010). A study on the relationship between some wild species of Chrysanthemum and cultivated chrysanthemum. MSc thesis. [Baoding(Hebei)]: Hebei Agricultural University, 1–58.

[ref60] Liu Q. J. (2013). Accumulation and antioxidant activity of phenolic substances in Opisthopappus taihangensis and related *Chrysanthemum indicum*. PhD dissertation. [Zhengzhou(Henan)]: Henan Agricultural University, 1–92.

[ref61] Liu Y. (2020). Comparative study on agronomic characters, chemical components and pharmacological effects of different chrysanthemum germplasm resources. MSc thesis. [Wuhan(Hubei)]: Hubei University of Chinese Medicine, 1–124.

[ref62] Liu H. Chen X. Chen H. Lu J. Chen D. Luo C.. (2021a). Transcriptome and metabolome analyses of the flowers and leaves of Chrysanthemum dichrum. Front. Genet. 12:716163. doi: 10.3389/fgene.2021.716163 34531898 PMC8438430

[ref63] Liu Y. Dai M. Bao W. Z. Huang B. S. Guo L. P. Liu D. H. (2021c). Characteristics of mineral elements in chrysanthemums from different origins in Macheng and their correlation with soil nutrients and effective components. China J. Chin. Mate. Med. 46, 281–289. doi: 10.19540/j.cnki.cjcmm.20200717.101 33645113

[ref64] Liu Y. Gong W. L. Bao W. Z. Guo L. P. Xu Y. Liu Y. M.. (2019). Establishment of *Chrysanthemum morifolium* HPLC fingerprint of Hubei and comparison of active components among varieties. China J. Chin. Mate. Med. 44, 3711–3717. doi: 10.19540/j.cnki.cjcmm.20190701.106 31602943

[ref65] Liu C. X. Guo D. A. Liu L. (2018). Quality transitivity and traceability system of herbal medicine products based on quality markers. Phytomedicine 44, 247–257. doi: 10.1016/j.phymed.2018.03.006, PMID: 29631807

[ref66] Liu H. B. Ma P. Xu L. J. Xiao P. G. (2021b). Informatics and big data: A new stage of pharmacophylogeny in medicinal plants. Mod. Chin. Med. 23, 1506–1511. doi: 10.13313/j.issn.1673-4890.20210901002

[ref67] Liu P. L. Wan Q. Guo Y. P. Yang J. Rao G. Y. (2012). Phylogeny of the genus Chrysanthemum L.: evidence from single-copy nuclear gene and chloroplast DNA sequences. PLoS One 7:e48970. doi: 10.1371/journal.pone.0048970, PMID: 23133665 PMC3486802

[ref68] Lu W. X. Hu X. Y. Wang Z. Z. Rao G. Y. (2022a). Hyb-Seq provides new insights into the phylogeny and evolution of the Chrysanthemum zawadskii species complex in China. Cladistics. doi: 10.1111/cla.12514, PMID: 35766338

[ref69] Lu Y. F. Li D. X. Zhang R. Zhao L. L. Qiu Z. Du Y.. (2022b). Chemical antioxidant quality markers of *Chrysanthemum morifolium* using a spectrum-effect approach. Front. Pharmacol. 13:809482. doi: 10.3389/fphar.2022.809482 35197853 PMC8859431

[ref70] Luo C. Chen D. L. Cheng X. Zhao H. E. Huang C. L. (2017). Genome size estimations in Chrysanthemum and correlations with molecular phylogenies. Genet. Resour. Crop. Evol. 64, 1451–1463. doi: 10.1007/s10722-016-0448-2

[ref71] Luo X. Y. Wang C. Dai S. L. Li B. Q. Liu Q. Q. Zhu J.. (2013). Genetic diversity of large-flowered Chrysanthemum cultivars revealed by ISSR analysis. Sci. Agri. Sin. 46, 2394–2402.

[ref72] Lybrand D. B. Xu H. Last R. L. Pichersky E. (2020). How plants synthesize pyrethrins: Safe and biodegradable insecticides. Trends Plant Sci. 25, 1240–1251. doi: 10.1016/j.tplants.2020.06.012, PMID: 32690362 PMC7677217

[ref73] Lyu L. Qin M. J. He D. X. Gu Y. H. (2008). Analyses of ISSR molecular marker and genetic relationship of different provenances of *Dendranthema morifolium*, D. indicum and D. nankingese. J. Plant Resour. Environ. 17, 7–12.

[ref74] Ma Y. P. Chen M. M. Wei J. X. Zhao L. Liu P. L. Dai S. L.. (2016). Origin of Chrysanthemum cultivars—Evidence from nuclear low-copy LFY gene sequences. Biochem. Syst. Ecol. 65, 129–136. doi: 10.1016/j.bse.2016.02.010

[ref75] Mandel J. R. Dikow R. B. Siniscalchi C. M. Thapa R. Watson L. E. Funk V. A. (2019). A fully resolved backbone phylogeny reveals numerous dispersals and explosive diversifications throughout the history of Asteraceae. Proc. Natl. Acad. Sci. U. S. A. 116, 14083–14088. doi: 10.1073/pnas.1903871116, PMID: 31209018 PMC6628808

[ref76] Mao C. Y. (2020). Study on quality evaluation standard of the flower of *Chrysanthemum morifolium* Ramat based on the correlation of ingredients and efficacy. [PhD dissertation. [Beijing]: China Academy of Chinese Medical Sciences, 1–246.

[ref77] Masuda Y. Nakano M. Kusaba M. (2022). The complete sequence of the chloroplast genome of Chrysanthemum rupestre, a diploid disciform capitula species of Chrysanthemum. Mitochondrial DNA B Resour. 7, 603–605. doi: 10.1080/23802359.2022.2057252 35386632 PMC8979539

[ref78] Matsuda K. (2012). Pyrethrin biosynthesis and its regulation in Chrysanthemum cinerariaefolium. Top. Curr. Chem. 314, 73–81. doi: 10.1007/128_2011_271, PMID: 22006239

[ref79] Mekapogu M. Vasamsetti B. M. K. Kwon O. K. Ahn M. S. Lim S. H. Jung J. A. (2020). Anthocyanins in floral colors: Biosynthesis and regulation in Chrysanthemum flowers. Int. J. Mol. Sci. 21, 6537. doi: 10.3390/ijms21186537, PMID: 32906764 PMC7554973

[ref80] Mérot C. (2020). Making the most of population genomic data to understand the importance of chromosomal inversions for adaptation and speciation. Mol. Ecol. 29, 2513–2516. doi: 10.1111/mec.15500, PMID: 32497331

[ref81] Nakano M. Hirakawa H. Fukai E. Toyoda A. Kajitani R. Minakuchi Y.. (2021). A chromosome-level genome sequence of Chrysanthemum seticuspe, a model species for hexaploid cultivated chrysanthemum. Commun. Biol. 4, 1167. doi: 10.1038/s42003-021-02704-y 34620992 PMC8497461

[ref82] Nie J. Xiao L. Zheng L. Du Z. Liu D. Zhou J.. (2019). An integration of UPLC-DAD/ESI-Q-TOF MS, GC-MS, and PCA analysis for quality evaluation and identification of cultivars of Chrysanthemi Flos (Juhua). Phytomedicine 59:152803. doi: 10.1016/j.phymed.2018.12.026 31005811

[ref83] Nishina A. Sato D. Yamamoto J. Kobayashi-Hattori K. Hirai Y. Kimura H. (2019). Antidiabetic-like effects of naringenin-7-O-glucoside from edible Chrysanthemum 'Kotobuki' and naringenin by activation of the PI3K/Akt pathway and PPARγ. Chem. Biodivers. 16:e1800434. doi: 10.1002/cbdv.201800434, PMID: 30462381

[ref84] Oberprieler C. Himmelreich S. Vogt R. (2007). A new subtribal classification of the tribe Anthemideae (Compositae). Willdenowia 37, 89–114. doi: 10.3372/wi.37.37104

[ref85] Park C. H. Chae S. C. Park S. Y. Kim J. K. Kim Y. J. Chung S. O.. (2015). Anthocyanin and carotenoid contents in different cultivars of Chrysanthemum (*Dendranthema grandiflorum* Ramat.) flower. Molecules 20, 11090–11102. doi: 10.3390/molecules200611090, PMID: 26083041 PMC6272539

[ref86] Park J. Choi S. Park S. Yoon J. Park A. Y. Choe S. (2019). DNA-free genome editing via ribonucleoprotein (RNP) delivery of CRISPR/Cas in lettuce. Methods Mol. Biol. 1917, 337–354. doi: 10.1007/978-1-4939-8991-1_25 30610648

[ref87] Peng A. Lin L. Zhao M. Sun B. (2019). Classification of edible chrysanthemums based on phenolic profiles and mechanisms underlying the protective effects of characteristic phenolics on oxidatively damaged erythrocyte. Food Res. Int. 123, 64–74. doi: 10.1016/j.foodres.2019.04.046, PMID: 31285013

[ref603] Peng Y. R. Shi L. Luo Y. H. (2006). Protective effect of the total flavones from Chrysanthemum on isoprenaline-induced myocardial ischemiain rats. Lishizhen Med. Materia Med. Res. 2006, 1131–1132.

[ref88] Poe S. Swofford D. L. (1999). Taxon sampling revisited. Nature 398, 299–300. doi: 10.1038/18592, PMID: 10192331

[ref604] Rajic A. Akihisa T. Ukiya M. Yasukawa K. Sandeman R. M. Chandler D. S.. (2001). Inhibition of trypsin and chymotrypsin by anti-inflammatory triterpenoids from Compositae flowers. Planta Med 67, 599–604. doi: 10.1055/s-2001-17350, PMID: 11582534

[ref89] Ryu J. Nam B. Kim B. R. Kim S. H. Jo Y. D. Ahn J. W.. (2019). Comparative analysis of phytochemical composition of gamma-irradiated mutant cultivars of *Chrysanthemum morifolium*. Molecules 24:3003. doi: 10.3390/molecules24163003, PMID: 31430944 PMC6720760

[ref90] Sawada Y. Sato M. Okamoto M. Masuda J. Yamaki S. Tamari M.. (2019). Metabolome-based discrimination of chrysanthemum cultivars for the efficient generation of flower color variations in mutation breeding. Metabolomics 15, 118. doi: 10.1007/s11306-019-1573-7 31451959

[ref91] Shao B. J. (2018). Preliminary study on germplasm resources innovation of forage species within Chrysanthemum in broad sense. MSc thesis. [Beijing]: Beijing Forestry University, 1–114.

[ref92] Shao Q. S. Guo Q. S. Li Y. C. Mao P. F. (2011). Quantitative analysis of morphological variation of medicinal Chrysanthemum germplasm resources. China J. Chin. Mate. Med. 36, 1261–1265. 21837961

[ref93] Shao Y. H. Sun Y. D. Li D. Chen Y. P. (2020). *Chrysanthemum indicum* L.: A comprehensive review of its botany, phytochemistry and pharmacology. Am. J. Chin. Med. 48, 871–897. doi: 10.1142/S0192415X20500421, PMID: 32431180

[ref94] Shen C. Z. Zhang C. J. Chen J. Guo Y. P. (2021). Clarifying recent adaptive diversification of the Chrysanthemum-group on the basis of an updated multilocus phylogeny of subtribe Artemisiinae (Asteraceae: Anthemideae). Front. Plant Sci. 12:648026. doi: 10.3389/fpls.2021.648026, PMID: 34122473 PMC8187803

[ref95] Song C. Liu Y. F. Song A. Dong G. Zhao H. B. Sun W.. (2018). The Chrysanthemum nankingense genome provides insights into the evolution and diversification of Chrysanthemum flowers and medicinal traits. Mol. Plant 11, 1482–1491. doi: 10.1016/j.molp.2018.10.003, PMID: 30342096

[ref96] Song Y. J. Xu L. J. Miao J. H. Xiao P. G. (2020). Research progress in *Chrysanthemum indicum*. Modern Chin. Med. 22, 1751–1756. doi: 10.13313/j.issn.1673-4890.20190722006

[ref97] Sun C. Q. Chen F. D. Fang W. M. Liu Z. L. Teng N. J. (2010a). Advances in research on distant hybridization of Chrysanthemum. Sci. Agri. Sin. 43, 2508–2517. doi: 10.3864/j.issn.0578-1752.2010.12.015

[ref98] Sun Q. L. Hua S. Ye J. H. Zheng X. Q. Liang Y. R. (2010b). Flavonoids and volatiles in *Chrysanthemum morifolium* Ramat flower from Tongxiang County in China. Afr. J. Biotechnol. 9, 3817–3821.

[ref99] Sun J. Wang Z. Chen L. Sun G. (2021). Hypolipidemic effects and preliminary mechanism of Chrysanthemum flavonoids, its main components luteolin and luteoloside in hyperlipidemia rats. Antioxidants. 10:1309. doi: 10.3390/antiox10081309, PMID: 34439559 PMC8389196

[ref100] Suo F. M. Chen S. L. Yu H. Xie C. X. Sun C. Z. (2011). Study on the origin suitability of four famous Chrysanthemums in China. Moderni. Trad. Chin. Med. Mater. Med.-World Sci. Technol. 13, 332–339.

[ref101] Tang F. P. (2009). Study on distant hybridization between Chrysanthemum and four related genera. PhD dissertation. [Nanjing(Jiangsu)]: Nanjing Agricultural University, 1–121.

[ref102] Tao J. H. (2017). Study on the effect mechanism of polysaccharides from non-medicinal parts of chrysanthemum on inflammatory bowel disease. PhD dissertation. [Nanjing(Jiangsu)]: Nanjing University of Chinese Medicine, 1–174.

[ref103] Tian Z. Jia H. Jin Y. Wang M. Kou J. Wang C.. (2019). Chrysanthemum extract attenuates hepatotoxicity via inhibiting oxidative stress in vivo and in vitro. Food Nutr. Res. 63:1667. doi: 10.29219/fnr.v63.1667, PMID: 31024225 PMC6475127

[ref104] Tian D. Yang Y. Yu M. Han Z. Z. Wei M. Zhang H. W.. (2020). Anti-inflammatory chemical constituents of Flos Chrysanthemi Indici determined by UPLC-MS/MS integrated with network pharmacology. Food Funct. 11, 6340–6351. doi: 10.1039/D0FO01000F, PMID: 32608438

[ref105] Tyagi S. Jung J. A. Kim J. S. Won S. Y. (2020). A comparative analysis of the complete chloroplast genomes of three Chrysanthemum boreale strains. PeerJ. 8:e9448. doi: 10.7717/peerj.9448, PMID: 32685287 PMC7337036

[ref107] van Lieshout N. van Kaauwen M. Kodde L. Arens P. Smulders M. J. M. Visser R. G. F.. (2022). De novo whole-genome assembly of Chrysanthemum makinoi, a key wild chrysanthemum. G3. 12, jkab358. doi: 10.1093/g3journal/jkab358 34849775 PMC8727959

[ref108] Wang L. Z. (2020). Preliminary identification of natural hybrids between *Chrysanthemum indicum* and Chrysanthemum vestitum. MSc thesis. [Taian(Shandong)]: Shandong Agricultural University, 1–56.

[ref109] Wang T. Y. Guo Q. S. Wang T. (2012). Karyotype analysis of 21 medicinal Chrysanthemum cultivation types. J. Nanjing Agri. Univ. 35, 13–18.

[ref110] Wang Y. J. Guo Q. S. Yang X. W. Xu W. B. Tao H. Y. (2008b). Characterization of chemical components of essential oil from flowers of *Chrysanthemum morifolium* produced in Anhui province. China J. Chin. Materia Med. 33, 2207–2211. 19166008

[ref111] Wang L. Hao D. C. Fan S. S. Xie H. T. Bao X. L. Jia Z. J.. (2022). N2O emission and nitrification/denitrification bacterial communities in upland black soil under combined effects of early and immediate moisture. Agriculture 12:330. doi: 10.3390/agriculture12030330

[ref112] Wang J. Liang Q. Zhao Q. Tang Q. Ahmed A. F. Zhang Y.. (2021b). The effect of microbial composition and proteomic on improvement of functional constipation by *Chrysanthemum morifolium* polysaccharide. Food Chem. Toxicol. 153:112305. doi: 10.1016/j.fct.2021.112305 34033886

[ref113] Wang D. Q. Liu S. J. Liang Y. M. (1999). A study on producing areas of Chinese flos Dendranthematis. China J. Chin. Mate. Medi. 24:573. 12205894

[ref114] Wang D. Q. Liu S. J. Liang Y. M. (2001). Research on medicinal taxa of Chinese chrysanthemum. J. Anhui College TCM. 20, 45–48.

[ref115] Wang Y. J. Su J. Yu J. J. Yan M. Q. Shi M. L. Huang Q. D.. (2021c). Buddleoside-rich *Chrysanthemum indicum* L. extract has a beneficial effect on metabolic hypertensive rats by inhibiting the enteric-origin LPS/TLR4 pathway. Fronti. Pharmacol. 12:755140. doi: 10.3389/fphar.2021.755140 PMC8532163 34690786

[ref116] Wang Y. S. Wang M. X. Han S. Zhang J. Shen X. Zhou J.. (2016). Differences in amino acid contents in fresh flowers and manufactured goods among four Chrysanthemum cultivars in Tongxiang City. Zhejiang Province. J. Anhui Agri. Univ. 43, 1024–1028. doi: 10.13610/j.cnki.1672-352x.20161205.011

[ref117] Wang Y. J. Yang X. W. Guo Q. S. (2008a). Studies on chemical constituents in Huangjuhua (flowers of *Chrysanthemum morifolium*). China J. Chin. Materia Med. 33, 526–530. 18536375

[ref118] Wang Z. Yuan Y. Hong B. Zhao X. Gu Z. (2021a). Characteristic volatile fingerprints of four Chrysanthemum teas determined by HS-GC-IMS. Molecules 26:7113. doi: 10.3390/molecules26237113 34885694 PMC8658894

[ref119] Watson L. E. Bates P. L. Evans T. M. Unwin M. M. Estes J. R. (2002). Molecular phylogeny of Subtribe Artemisiinae (Asteraceae), including Artemisia and its allied and segregate genera. BMC Evol. Biol. 2:17. doi: 10.1186/1471-2148-2-17, PMID: 12350234 PMC130036

[ref120] Wei M. Zhang Y. J. Wang T. Guo Q. S. Zou Q. J. Chen F. R.. (2021). Correlations between content of linarin in Chrysanthemum indicum and climatic factors in habitats. China J. Chin. Materia Med. 46, 2167–2172. doi: 10.19540/j.cnki.cjcmm.20210320.104 34047117

[ref121] Won S. Y. Kwon S. J. Lee T. H. Jung J. A. Kim J. S. Kang S. H.. (2017). Comparative transcriptome analysis reveals whole-genome duplications and gene selection patterns in cultivated and wild Chrysanthemum species. Plant Mol. Biol. 95, 451–461. doi: 10.1007/s11103-017-0663-z, PMID: 29052098 PMC5727146

[ref122] Wu G. S. (2007). Study on the genetic relationship between some Chrysanthemum and Ajania plants. MSc thesis. [Nanjing (Jiangsu)]: Nanjing Agricultural University, 1–101.

[ref123] Wu X. B. (2014). Preliminary study on distant hybridization of Chrysanthemum sensu lato (VII). MSc thesis. [Beijing]: Beijing Forestry University, 1–62.

[ref124] Wu X. Sun Y. Shen X. Wang Z. (2015). Study on combined effects of chemical components for different flowers blossoming degree of yellow medicinal *Chrysanthemum morifolium* from Zhejiang. China J. Chin. Mate. Med. 40, 3174–3178. 26790287

[ref125] Xiao Y. (2019). Research on nutritional and functional properties of Chrysanthesum mongolicum tea and its application in whey tea beverage. MSc thesis. [Hohhot (Inner Mongolia)]: Inner Mongolia Agricultural University, 1–62.

[ref126] Xie F. (2016). Preliminary study on distant hybridization of Chrysanthemum (IX): Germplasm utilization of Hippolytia and other genera. MSc thesis. [Beijing]: Beijing Forestry University, 1–82.

[ref127] Xie Y. Qu J. Wang Q. Wang Y. Yoshikawa M. Yuan D. (2012). Comparative evaluation of cultivars of *Chrysanthemum morifolium* flowers by HPLC-DAD-ESI/MS analysis and antiallergic assay. J. Agri. Food Chem. 60, 12574–12583. doi: 10.1021/jf304080v, PMID: 23214422

[ref128] Xiong Y. X. (2014). Research on the germplasm resources of Fubaiju and the selection of excellent lines. MSc thesis. [Wuhan(Hubei)]: Hubei University of Chinese Medicine, 1–125.

[ref129] Xu W. B. Guo Q. S. Li Y. N. Wang T. (2005). Comparative study on internal quality of various *Chrysanthemum morifolium*. China J. Chin. Mate. Med. 30, 1645–1648. 16400937

[ref130] Xu W. B. Guo Q. S. Wang C. L. (2006). RAPD analysis of genetic diversity of medicinal *Chrysanthemum morifolium*. China J. Chin. Mate. Medi. 2006, 18–21. 16548159

[ref131] Xu M. Jiang Y. Chen S. Chen F. Chen F. (2021). Herbivory-induced emission of volatile terpenes in *Chrysanthemum morifolium* functions as an indirect defense against *Spodoptera litura* larvae by attracting natural enemies. J. Agri. Food Chem. 69, 9743–9753. doi: 10.1021/acs.jafc.1c02637, PMID: 34465092

[ref132] Xue H. Jiang Y. Zhao H. Köllner T. G. Chen S. Chen F. D.. (2019). Characterization of composition and antifungal properties of leaf secondary metabolites from thirteen cultivars of *Chrysanthemum morifolium* Ramat. Molecules 24:4202. doi: 10.3390/molecules24234202, PMID: 31756889 PMC6935761

[ref133] Xue G. M. Li X. Q. Chen C. Chen K. Wang X. B. Gu Y. C.. (2018). Highly oxidized guaianolide sesquiterpenoids with potential anti-inflammatory activity from *Chrysanthemum indicum*. J. Nat. Prod. 81, 378–386. doi: 10.1021/acs.jnatprod.7b00867, PMID: 29400471

[ref134] Xue L. M. Qin X. M. Guo J. G. (2007). Comparative study on main components of *Chrysanthemum morifolium* introduced in Ruicheng in Shanxi Province. Chin. Trad. Herb. Drug. 2007, 751–754.

[ref135] Xue G. M. Xue J. F. Zhao C. G. Zhao Z. Z. Zhi Y. L. Du K.. (2021). 1,10-seco guaianolide-type sesquiterpenoids from *Chrysanthemum indicum*. J. Asian Nat. Prod. Res. 23, 877–883. doi: 10.1080/10286020.2020.1787388, PMID: 32603195

[ref136] Yan H. Y. Liu Y. Xu Y. Fang Y. Guo L. P. Liu D. H. (2021). Analysis and evaluation of mineral elements of *Chrysanthemum morifolium* for medicinal and tea use of different germplasm resources. China J. Chin. Mate. Med. 46, 272–280. doi: 10.19540/j.cnki.cjcmm.20201023.101 33645112

[ref137] Yang L. Aobulikasimu N. Cheng P. Wang J. H. Li H. (2017a). Analysis of floral volatile components and antioxidant activity of different varieties of *Chrysanthemum morifolium*. Molecules 22:1790. doi: 10.3390/molecules22101790 PMC6151829 29065520

[ref138] Yang C. F. Dong C. M. Xing B. Xia W. Tian Y. J. (2018a). Study on quality of *Chrysanthemum morifolium* from different habitats. Mod. Chin. Med. 20, 716–720. doi: 10.13313/j.issn.1673-4890.20170519008

[ref139] Yang P. F. Feng Z. M. Yang Y. N. Jiang J. S. Zhang P. C. (2017b). Neuroprotective caffeoylquinic acid derivatives from the flowers of *Chrysanthemum morifolium*. J. Nat. Prod. 80, 1028–1033. doi: 10.1021/acs.jnatprod.6b01026 28248102

[ref140] Yang W. Glover B. J. Rao G. Y. Yang J. (2006). Molecular evidence for multiple polyploidization and lineage recombination in the *Chrysanthemum indicum* polyploid complex (Asteraceae). New Phytol. 171, 875–886. doi: 10.1111/j.1469-8137.2006.01779.x, PMID: 16918557

[ref141] Yang X. W. Han M. H. Tao H. Y. Wang Z. A. Yang Z. Xiao S. Y. (2007). GC-MS analysis of essential oil from anthodiums of *Chrysanthemum morifolium* processed by microwave-airflow and steam calefaction. China J. Chin. Mate. Medi. 32, 227–230. doi: 10.1021/acs.jnatprod.6b01026 17432145

[ref142] Yang Y. J. Jiang Z. F. Guo J. Yang X. Xu N. Chen Z.. (2018b). Transcriptomic analyses of *Chrysanthemum morifolium* Ramat under UV-B radiation treatment reveal variations in the metabolisms associated with bioactive components. Indus. Crop Prod. 124, 475–486. doi: 10.1016/j.indcrop.2018.08.011

[ref143] Yang P. F. Yang Y. N. Feng Z. M. Jiang J. S. Zhang P. C. (2019). Six new compounds from the flowers of Chrysanthemum morifolium and their biological activities. Bioorg. Chem. 82, 139–144. doi: 10.1016/j.bioorg.2018.10.007, PMID: 30321776

[ref144] Zhang R. D. (2008). The Chrysanthemum culture research in ancient China. PhD dissertation. [Nanjing(Jiangsu)]: Nanjing Normal University, 16–25.

[ref605] Zhang L. J. Dai S. L. (2009). Research advance on germplasm resources of Chrysanthemum morifolium. Chin. Bull. Bot. 44, 526–535. doi: 10.3969/j.issn.1674-3466.2009.05.002, PMID: 10192331

[ref145] Zhang E. X. Fang L. Zhang J. Yu L. J. Xiao X. (2000). The antioxidant activity research of Chrysanthemum extract. Food Sci. 21, 6–9.

[ref146] Zhang N. He Z. He S. Jing P. (2019). Insights into the importance of dietary chrysanthemum flower (*Chrysanthemum morifolium* cv. Hangju)-wolfberry (*Lycium barbarum* fruit) combination in antioxidant and anti-inflammatory properties. Food Res. Int. 116, 810–818. doi: 10.1016/j.foodres.2018.09.015, PMID: 30717012

[ref147] Zhang W. Jiang Y. Chen S. Chen F. D. Chen F. (2021b). Concentration-dependent emission of floral scent terpenoids from diverse cultivars of Chrysanthemum morifolium and their wild relatives. Plant Sci. 309:110959. doi: 10.1016/j.plantsci.2021.110959 34134850

[ref148] Zhang K. Jiang Y. Zhao H. Köllner T. G. Chen S. Chen F. D.. (2020). Diverse terpenoids and their associated antifungal properties from roots of different cultivars of *Chrysanthemum morifolium* Ramat. Molecules 25:2083. doi: 10.3390/molecules25092083, PMID: 32365690 PMC7248984

[ref149] Zhang W. Xu H. Duan X. Hu J. Li J. Zhao L.. (2021a). Characterizing the leaf transcriptome of Chrysanthemum rhombifolium (Ling et C. Shih), a drought resistant, endemic plant from China. Front. Genet. 12:625985. doi: 10.3389/fgene.2021.625985 33643389 PMC7906282

[ref150] Zhang Z. Zhang Y. Wang L. Cui T. Wang Y. Chen J.. (2022). On-line screening of natural antioxidants and the antioxidant activity prediction for the extracts from flowers of *Chrysanthemum morifolium* ramat. J. Ethnopharmacol. 294:115336. doi: 10.1016/j.jep.2022.115336, PMID: 35568113

[ref151] Zhao H. B. (2007). Phylogeny of tribe Anthemideae (Asteraceae) from East Asia and intergeneric cross between Dendranthema×grandiflorum (Ramat.) Kitam. and Ajania pcifica (Nakai) K. Bremer & Humphries. PhD dissertation. [Nanjing(Jiangsu)]: Nanjing Agricultural University, 1–181.

[ref152] Zhao S. H. (2015). Analysis of nutrient composition of six kinds of chrysanthemum. MSc thesis. [Kaifeng(Henan)]: Henan University, 1–81

[ref153] Zhao H. B. Chen F. D. Chen S. M. Wu G. S. Guo W. M. (2010). Molecular phylogeny of Chrysanthemum, Ajania and its allies (Anthemideae, Asteraceae) as inferred from nuclear ribosomal ITS and chloroplast trnL-F IGS sequences. Plant Syst. Evol. 284, 153–169. doi: 10.1007/s00606-009-0242-0

[ref154] Zhao H. B. Chen F. D. Guo W. M. Tang F. P. Fang W. M. (2008). Preliminary study on hybrid compatibility between Chrysanthemum and some genera of Anthemideae. J. Nanjing Agri. Univ. 2008, 139–143.

[ref157] Zheng C. P. (2015). Isolation, purification, structure identification and bioactivity of chrysanthemum polysaccharide. MSc thesis. [Nanchang(Jiangxi)]: Nanchang University, 1–107

[ref158] Zhou J. (2009). Studies on the problem of origin of Chinese garden chrysanthemum. PhD dissertation. [Beijing]: Beijing Forestry University, 1–148.

[ref159] Zhou L. Liu Q. Hong G. Song F. Zhao J. Yuan J.. (2019a). Cumambrin A prevents OVX-induced osteoporosis via the inhibition of osteoclastogenesis, bone resorption, and RANKL signaling pathways. FASEB J. 33, 6726–6735. doi: 10.1096/fj.201800883RRR, PMID: 30807230

[ref160] Zhou H. P. Ren M. X. Guan J. Q. Liu Y. L. Xiong Y. X. Zhong Q. F.. (2019b). Research progress on chemical constituents and pharmacological effects of Chrysanthemum morifolium and predictive analysis on quality markers. Chin. Trad. Herb. Drug. 50, 4785–4795.

[ref162] Zhou Z. Xian J. Wei W. Xu C. Yang J. Zhan R.. (2021). Volatile metabolic profiling and functional characterization of four terpene synthases reveal terpenoid diversity in different tissues of *Chrysanthemum indicum* L. Phytochemistry 185:112687. doi: 10.1016/j.phytochem.2021.112687, PMID: 33588133

[ref163] Zhuo F. F. Zhang C. Zhang H. Xia Y. Xue G. M. Yang L.. (2019). Chrysanthemulide A induces apoptosis through DR5 upregulation via JNK-mediated autophagosome accumulation in human osteosarcoma cells. J. Cellu. Physiol. 234, 13191–13208. doi: 10.1002/jcp.27991, PMID: 30556589 PMC13483265

[ref164] Zou Q. Guo Q. Wang T. Chen J. Yang F. Yang C. (2022). Comparison of metabolome characteristics and screening of chemical markers in *Chrysanthemum indicum* from different habitats. Physiol. Mol. Biol. Plants 28, 65–76. doi: 10.1007/s12298-022-01137-z, PMID: 35221572 PMC8847665

