# Supplementary figures and images for: The genus Chrysanthemum: Phylogeny, biodiversity, phytometabolites, and chemodiversity
Source: Front Plant Sci. 2022 Aug 11;13:973197. doi: 10.3389/fpls.2022.973197 (PMC9403765; doi:10.3389/fpls.2022.973197)

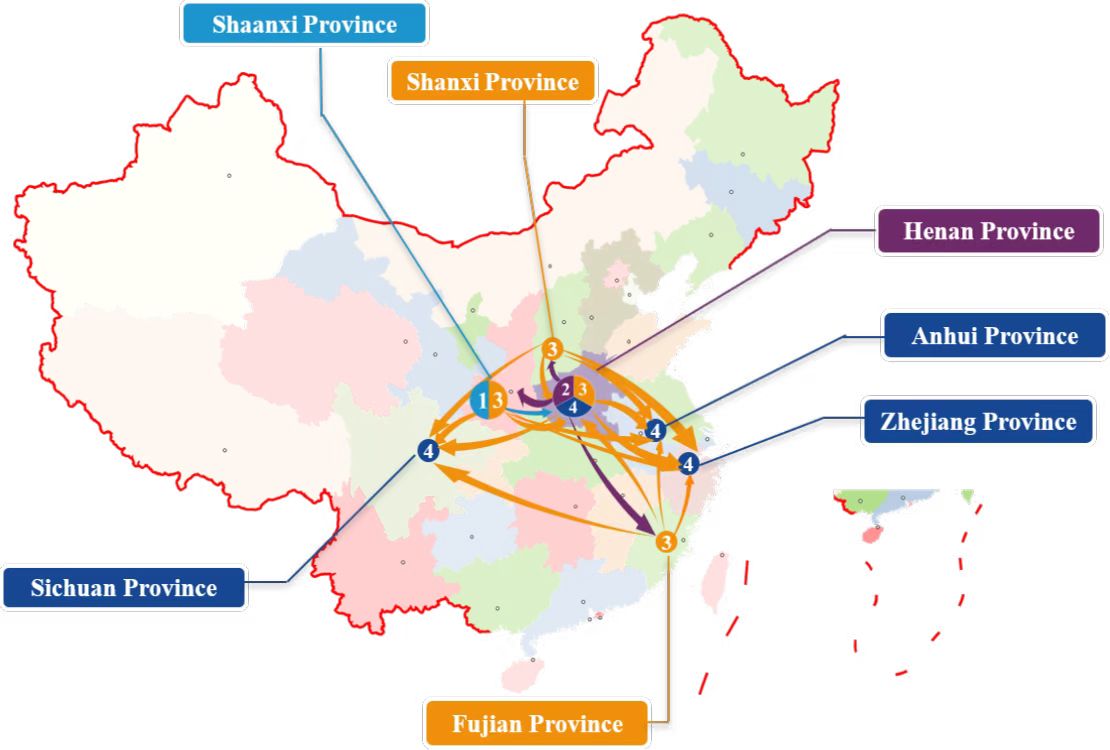

Supplement: Supplementary file 2 [file Image_1.JPEG]
